# Investigating the evolution of large meiotic rings of multiple X and Y sex chromosomes in two *Leptodactylus* frog species (Anura, Leptodactylidae)

**DOI:** 10.1038/s42003-025-09151-z

**Published:** 2025-11-21

**Authors:** Jhon Alex Dziechciarz Vidal, Deborah Charlesworth, Wen-Juan Ma, Qi Zhou, Ricardo Utsunomia, Anderson José Baia Gomes, Amanda Bueno da Silva, Fábio Porto-Foresti, Thomas Liehr, Marcelo de Bello Cioffi

**Affiliations:** 1 Laboratory of Evolutionary Cytogenetics, Department of Genetics and Evolution, Federal University of São Carlos, São Carlos, SP Brazil; 2 Institute of Ecology and Evolution, Ashworth Laboratories, King’s Buildings, University of Edinburgh, Edinburgh, UK; 3 Research group of Ecology, Evolution and Genetics, Biology Department, Vrije Universiteit Brussel, Brussels, Belgium; 4 Life Sciences Institute, Zhejiang University, Hangzhou, Zhejiang China; 5 Faculdade de Ciências, UNESP, Bauru, São Paulo Brazil; 6 Laboratório de Biologia Molecular, Evolução e Microbiologia, Instituto Federal do Pará, Abaetetuba, Brazil; 7 Jena University Hospital, Friedrich Schiller University, Institute of Human Genetics, Jena, Germany

**Keywords:** Evolutionary biology, Evolutionary genetics

## Abstract

A few species have evolved multiple sex chromosome systems with more than two Xs or Ys due to sex chromosome-autosome translocations. Among vertebrates, frogs (Anura) have the highest known number of such neo-sex chromosome systems, making them interesting for studying how such systems evolve. In this work, we investigated two *Leptodactylus* species, *L. pentadactylus* (LPE) and *L. paraensis* (LPA), with large ring multivalents in male meiosis, using genomic and cytogenetic investigation of repetitive DNA sequences, including satellite DNAs (satDNAs), and transposable elements (TEs). SatDNA mapping identify individual chromosomes in the LPE ring, and morphologies suggest that all chromosomes are shared with the LPA ring although a common ring origin is not firmly supported. In situ mapping suggests recent satDNA accumulation in subtelomeric regions since the split from the outgroups, likely unrelated to the translocations that created sex-linkage, which probably involved breaks in the pericentromeric regions.

## Abstract (web summary)

Comparative analysis of repetitive DNA in two Amazonian frogs reveals multiple sex chromosome systems and ring multivalents, clarifying chromosomal composition and evolution in *Leptodactylus pentadactylus* and *L. paraensis*.

## Introduction

Sex chromosomes have evolved independently in many groups of organisms, and the degrees of differentiation between members of the pairs vary greatly across species (reviewed in ref. ^1^). In animals, most species have XX/XY or ZZ/ZW sex chromosome systems (male and female heterogamety, respectively, and sometimes the Y or W lacks (almost) all functional genes, termed “highly degenerated”). A minority have multiple sex chromosome systems reflecting translocations between autosomes and one or both members of a pair of ancestral sex chromosomes, or fusions, which involve translocations when one arm is small and heterochromatic^2–4^. Reciprocal translocations are expected to greatly reduce fertility in heterozygotes^2^ and many translocations will thus fail to spread in populations unless selection favours the new arrangement^5^. Nevertheless, many systems with more than one X or Y chromosome, include rodents^6^, monkeys^7^, fish (reviewed in ref. ^4^) and plants^8^; the same can be seen for more than one Z chromosome in Lepidoptera, with ZZ/ZW systems^9–11^.

A few species have evolved systems involving more than one autosome as well as the sex chromosome pair. All known multiple systems with meiotic chains or rings are derived from XY systems^12–15^, including monotreme (non-Eutherian) mammals, the platypus *Ornithorhynchus anatinus* and the short-beaked echidna *Tachyglossus aculeatus*, with X_1_–X_5_/Y_1_–Y_5_ and X_1_–X_5_/Y_1_–Y_4_ respectively, systems with multivalent chains of sex chromosomes in male meiosis^12,16,17^. The evolution of these systems is not yet well understood, although it is clear that the components of large regions are fully Y-linked, as they have become genetically degenerated in both species and have lost most genes carried on their X counterparts^18^. Studying a younger system may provide information about the time course of degeneration and possibly the reason for these systems’ formation.

Among other vertebrate clades, frogs include species with chromosome chains or ring multivalents; three cases have currently been described. Males of the smoky jungle frog *Leptodactylus pentadactylus* (hereafter denoted by LPE) have the largest multiple sex chromosome system so far identified in any vertebrate, a ring of 12 chromosomes that are largely unpaired in male meiosis (an X1–X6/Y1–Y6 system, see refs. ^13,15^, though some populations of this species have a ring of 10^19^. Large meiotic rings have been described in two other frogs distantly related to *Leptodactylus*: the clay robber *Haddadus binotatus* (Craugastoridae)^20^ and the Taiwanese frog *Odorrana swinhoana*^21^, with rings of 8 and 6 chromosomes, respectively. The two *Leptodactylus* species studied here, LPE and *L. paraensis* (abbreviated to LPA) diverged from the other two frog species approximately 86 and 150 MYA, respectively^22^; thus, meiotic rings most likely emerged independently in these three frog lineages.

As so few species have multi-chromosomal chains or rings with more than four sex chromosomes (reviewed in ref. ^12^), frogs might have an unusual tendency to undergo multiple rearrangements involving the sex chromosomes, perhaps because their chromosomes have suitable properties (see below). Frogs might thus be suitable for studying the rates of appearance of X- and/or Y-autosomal translocations and meiotic rings, and, if they show an increased incidence compared with other organisms, what factors lead to this. In the case of rings involving the sex chromosomes, translocations might, for example, be an evolutionary response to a situation in which recombination is disfavoured. An autosomal sexually antagonistic polymorphism might become fully sex-linked via mutual translocation with part of the sex-linked region. However, testing the hypothesis that such selection favours an observed lack of recombination between the sex chromosomes is notoriously difficult.

Before considering factors that might favour reciprocal translocations, it is therefore important to consider the genomic and ecological contexts that affect the probability of such rearrangements establishing. Genomic factors include characteristics that (i) increase the probability of a reciprocal translocation arising or (ii) might decrease the deleterious consequences of chromosomal breaks (such as frequent unbalanced gametes or disrupting the effects of upstream regulatory sequences on the expression of genes) so that a translocation is more likely to be able to increase in frequency in a population.

Category (i) has been the subject of numerous studies testing for regions, particularly prone to chromosomal breakage (fragile regions), in genomes. These regions are often referred to as “evolutionary breakpoint regions” (EBRs) to denote their tendency to experience chromosome breakage in independent lineages^23,24^. Analysis of numerous eukaryotic genomes indicates that EBRs and fragile sites tend to be regions with particularly high repetitive densities, such as pericentromeric and subtelomeric regions (reviewed in ref. ^23^). This might be expected, since repetitive sequences can produce DNA structures, such as hairpins, which may facilitate chromosomal rearrangements and can recombine “ectopically” with similar sequences in other genome regions^23,24^. On the other hand, open chromatin, which makes breaks more likely, is associated with high densities of active genes and high recombination rates. Such genomic properties can account for convergent breakpoints in independent lineages. In the case of species with reciprocal translocations creating rings, the breakpoints are most likely to be in highly repetitive middle regions of metacentric chromosomes in the ancestors. However, regions with highly repetitive sequences may appear in new genomic locations, including neo-centromeres (as reviewed in ref. ^25^), or be lost from existing locations, causing turnovers of repeat content, especially in rarely recombining regions^26^, so different rearrangement breakpoint sites are possible in related species^23^.

Category (ii) includes genomic factors that, rather than increasing the probability that rearrangements arise, decrease their deleterious consequences, making them more likely to persist once they arise. This category includes weak purifying selection, predicting that EBRs will be mainly in permanent genomic features such as intergenic regions (as reviewed in ref. ^23^). Two other important factors of this kind are the localization of crossovers mainly to chromosome tips and alternating segregation of adjacent centromeres in the multivalent in meiosis, which reduces the frequency of unbalanced gametes. Importantly, if the middle chromosome regions that rarely cross over are repeat-rich in the ancestral species, this characteristic will usually be inherited by the rings. Conversely, repeat-richness (a category (i) characteristic causing a high origination rate of rearrangements) correlates with low recombination rates and low gene densities, because the absence of recombination enables rearrangements to occur by ectopic exchanges without inducing adverse effects in meiosis. Both categories (i) and (ii) must be considered when trying to explain why certain species have evolved sex chromosome multivalents.

A third category must also be considered. Category (iii) includes characteristics affecting the probability that individuals in a population will be heterozygous for a translocation. A newly arisen translocation will initially be almost exclusively heterozygous, and such heterozygotes often suffer the well-known disadvantages of unbalanced gamete production (reviewed in ref. ^2,27^). In small and isolated populations, however, genetic drift may allow such a rearrangement to reach a high enough frequency to avoid being lost and sometimes to fix ref. ^28^. Inbreeding similarly allows translocations to spread in a population, simply because heterozygotes are rare, and indeed, there is evidence for higher chromosome rearrangement rates in inbreeders (reviewed in ref. ^29^). A previous study of one *Leptodactylus* frog species that has a sex chromosome multivalent, LPE, showed that segregation of adjacent centromeres alternates in male meiosis, a category (ii) factor that facilitates the production of balanced gametes^30^. We return to the other categories in the Discussion section, but here we focus on the genomic category (i) factor using combined genome sequencing and cytogenetic in situ hybridization analysis of several species with sex chromosome multivalent ring systems. The goals were (a) to describe repetitive satellite DNA sequences that might represent EBRs involved in causing chromosome breaks, together with some preliminary results from transposable elements; (b) evaluate whether the translocations evolved independently in these lineages or evolved in a common ancestor, and whether the evolution of the large chromosomal ring multivalents caused the component chromosomes to become partially or completely sex-linked; and (c) to describe changes since the rings evolved. We analysed six *Leptodactylus* species, of which only LPE and LPA have ring sex chromosomes, which have not previously been studied in LPA. Our study included a new LPE population, which revealed a different number of chromosomes in the ring, showing that new translocations are still possible in this species complex^13,31^.

## Results

### Evolutionary relationships among the species studied

**Fig. 1:**
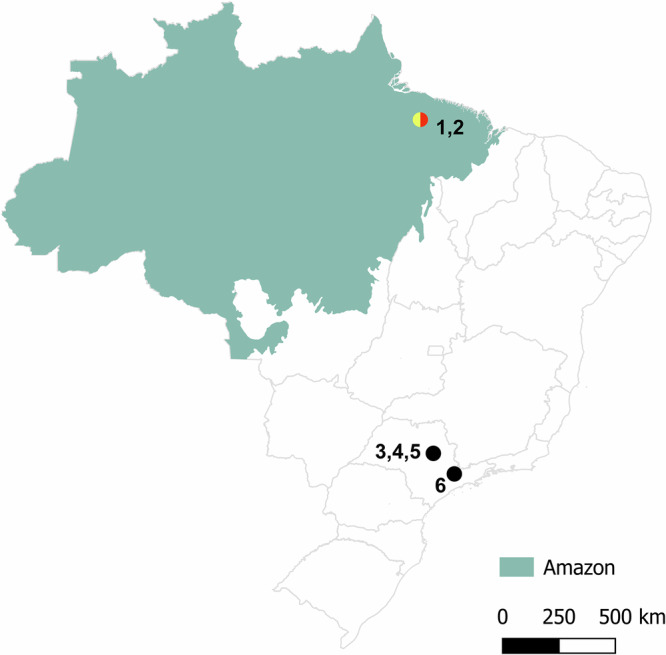
Map of Brazil showing the Amazon region and the sampling sites of the frog species studied here. The red, yellow and black dots respectively represent the newly studied populations with meiotic rings (LPE with rings of 10 chromosomes, LPA with a ring of 8 chromosomes) and of other species with no rings (numbers representing the different samples are in Table 1.

**Fig. 2:**
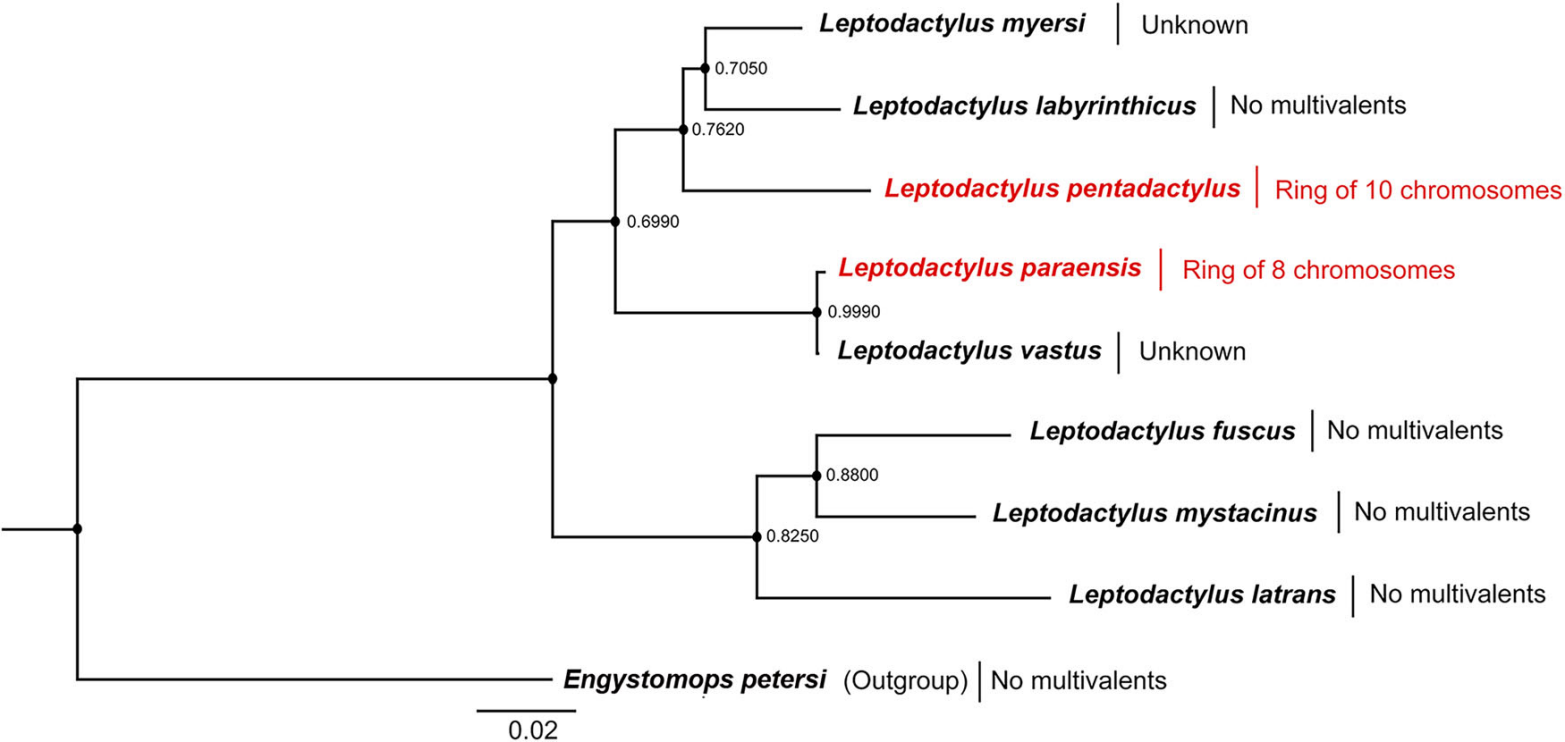
Maximum likelihood tree using partial sequences of the mitochondrial 16S RNA gene from several *Leptodactylus* species, rooted with an outgroup *Engystomops petersi.* The two species with meiotic rings (LPA and LPE) are indicated by red font. The scale bar represents nucleotide divergence per site (based on all sites, as this is not a coding sequence), and values at the nodes indicate bootstrap support values.

Here, we analysed six *Leptodactylus* species. Two, *Leptodactylus pentadactylus* (LPE) and *Leptodactylus paraensis* (LPA), were collected in sympatry in the Amazonian rainforest in northern Brazil, approximately 1800 km from the site previously sampled ref. ^13^. The four other species investigated, *L. fuscus, L. mystacinus, L. latrans*, and *L. labyrinthicus* (Table 1), were sampled from different populations that had not previously been studied (Fig. 1). Figure 2 shows a phylogenetic tree of these species.

**Table 1:** *Leptodactylus* species and populations newly studied here, including the numbers of individuals studied in male meiosis, and the geographic coordinates of the sampling sites

Species names, with the numbers used in Fig. 1 (and abbreviated names, when used)	Presence of rings in male meiosis	Number of individuals studied	GPS coordinates of the collection localities
1- *Leptodactylus pentadactylus* (LPE)	Yes	♀ 6/8♂	1°41'06.0“S 48°37'50.0“W
2- *Leptodactylus paraensis* (LPA)	Yes	4♂	1°41'06.0“S 48°37'50.0“W
3- *Leptodactylus fuscus*	No	♀ 5/5♂	21°55'41.6“S 47°53'14.4“W
4- *Leptodactylus mystacinus*	No	♀ 3/3♂	21°55'47.9“S 47°53'22.0“W
5- *Leptodactylus labyrinthicus*	No	♀ 2/2♂	21°58'02.6“S 47°53'03.7“W
6- *Leptodactylus latrans*	No	♀ 1/1♂	22°54'53.5“S 45°57'37.2“W

### Cytogenetic analyses

**Fig. 3:**
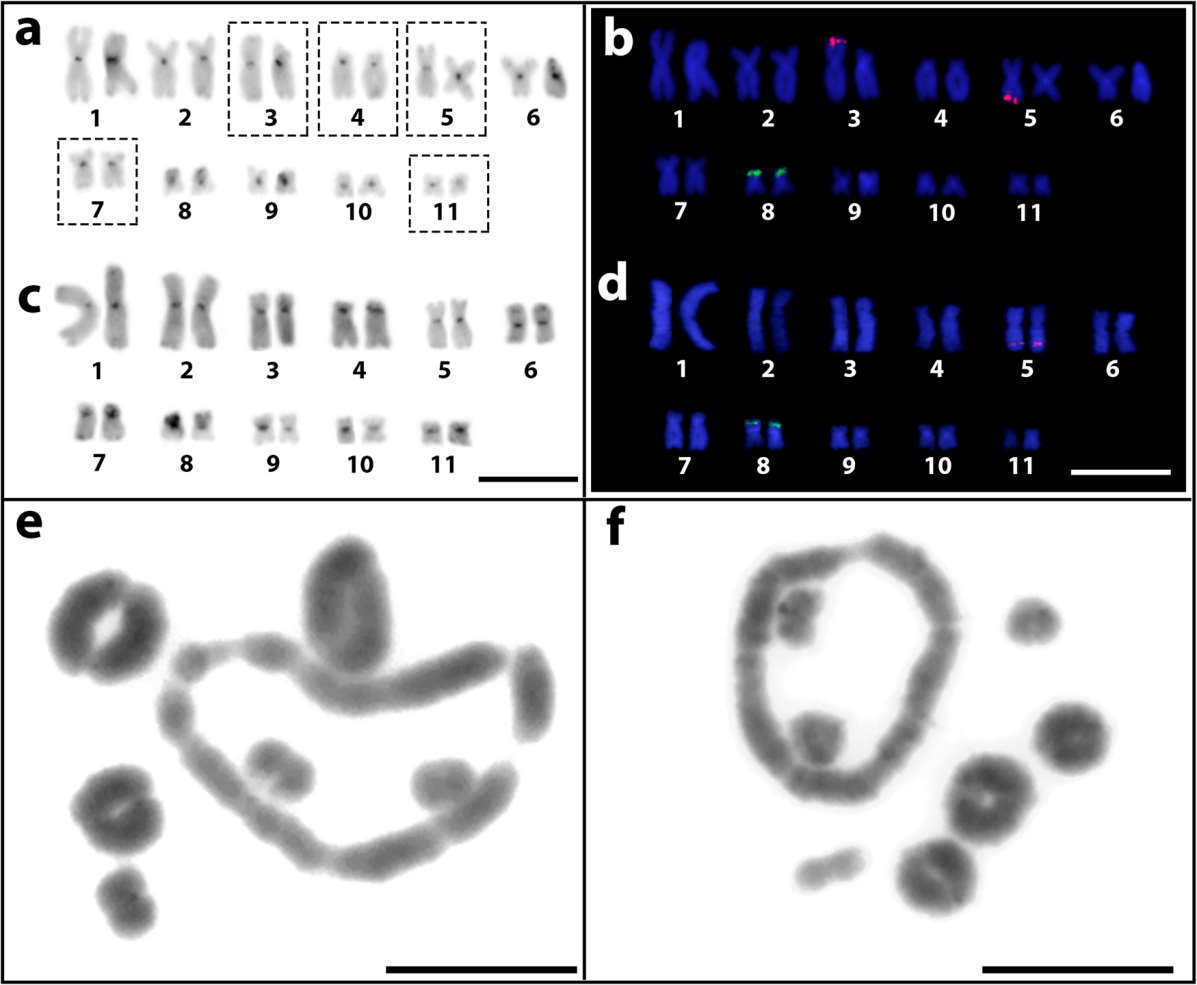
Karyotype and meiotic configurations in males and females of LPE and LPA. Karyotypes of male (**a** and **b**) and female (**c** and **d**) of LPE analysed by C-banding to show heterochromatic regions, including centromeric regions in the middle of each chromosome (**a** and **c**) and FISH with two probes, 18S and 5S rDNA, in green and red, respectively (**b** and **d**). In metaphase I, LPE has six bivalents and a ring of 10 chromosomes (**e**), and LPA has a ring of eight chromosomes and seven bivalents (**f**). Bars = 10 μm.

Out of the ~120 *Leptodactylus* frog species, 45 have been analysed cytogenetically, and their karyotypes usually display 2*n* = 22 metacentric or sub-metacentric chromosomes, including all six species analysed here (Fig. 3, Supplementary Fig. 1). Corroborating previous descriptions^15,31,32^, chromosome morphologies in mitosis are conserved in the different species analysed, with seven larger chromosomes and four smaller ones. Individual chromosome lengths in mitotic preparations of males of *L. pentadactylus* and *L. fuscus* show that relative total lengths are highly similar in the two species, with an *r*^2^ value for a linear regression of 97%, though the arm ratios vary (Supplementary Table 1 and Supplementary Fig. 2). C-positive heterochromatin is detected in the pericentromeric regions of all chromosomes of *L. paraensis* and *L. pentadactylus* during mitosis (Fig. 3 and Supplementary Fig. 3), corroborating previous results described in refs. ^15,31^, although in some species, blocks are also seen in other locations^15,31,33^.

In male meiosis (metaphase I and II), four species (*L. fuscus, L. mystacinus, L. latrans* and *L. labyrinthicus*) have no multivalents (Supplementary Fig. 2), as previously reported for two of these species and two other species^15^. In all species studied, the bivalents form rings during male meiosis, and the same is seen in both species with multivalents (Fig. 3e, f and Supplementary Fig. 2). Crossover localization to chromosome tips is therefore probably ancestral in this genus. Few studies have examined meiosis (Supplementary Table 2), and it is thus unclear how many species have systems with chromosomal chains or rings.

In mitotic preparations, the chromosomes of both sexes in LPE (Fig. 3a, c) closely resemble those of *L. fuscus*^15^. In addition to the heterochromatic blocks in the middle regions of chromosomes of all species studied (see above), blocks were also visible in the terminal regions of LPE pairs 6, 7, and 8 in both sexes (Fig. 3a, c). LPE populations with rings of 12 chromosomes in male meiosis have previously been documented^13,15^, which must involve at least four X- and Y-autosome reciprocal translocations, and we now describe a population from a nearby location (Fig. 1) with only 10 chromosomes in the ring (including about 42% of the genome, based on the lengths in Fig. 3a, b) plus six bivalents, similar to the state described by ref. ^19^. In mitosis, all five males investigated showed heteromorphism for chromosomes 3 and 5, pair (Fig. 3a, b). Specifically, the chromosome 3 and 5 short arms are longer than those of the non-male-specific homologues, as expected if these arms have stopped recombining and started accumulating repeats. One or other of these heteromorphic chromosomes may represent the ancestral XY pair, as discussed below. For LPA, despite considerable effort, mitotic preparations were unsuccessful, and it is therefore unclear whether chromosome 3 or 5 is heteromorphic in this species also. In male meiosis, LPA shows a ring of 8 (about 30% of the genome, based on the chromosome lengths in male meiotic karyotypes, plus seven bivalents (Fig. 3f). Both the rings and univalents again segregate in meiosis II, resulting in cells with the expected *n* = 11 (Supplementary Fig. 3). Schematic ideograms of the meiotic rings in LPE and LPA are shown below, after descriptions of the identification of some of the component chromosomes.

### Repetitive DNA composition of *L. pentadactylus* and *L. fuscus* and characterization of LpeSatDNAs

We first studied the overall repetitive contents of these frog genomes. For a male of the distant outgroup species, *L. fuscus*, de novo TE annotation using the RepeatModeler2 pipeline estimated that nearly 58.5% of the genome consists of repeats, mostly TEs (Supplementary Figs. 4 and 5), similar to estimates from other Leptodactylidae (63%) and Hylidae (65%)^34^. Based on the TEs ascertained in the *L. fuscus* genome assembly (GCA_031893025.1), analysis of the unassembled very low-coverage Illumina short reads from LPE (see the “Methods” section) using the DNApipeTE software yielded total repeat content estimates around 50%, including satellite sequences. Although satellite sequences form only around 2% of the total amount of repeats (Supplementary Fig. 5), we analyse them further below, because they can help identify chromosome regions of potential interest (see below) and individual chromosomes.

Most of the major TE classes were identified in both species, with DNA transposons being common (Supplementary Fig. 4), but most being unknown or non-annotated types. To initiate understanding of their ages, we analysed repeat landscapes using RepeatModeler (see the “Methods” section). For *L. fuscus*, this suggested, unsurprisingly, that TEs are active in this species, but that some insertions have high divergence values from their consensus sequences, as is usual in most genomes analysed, suggesting the presence of some old insertions (Supplementary Fig. 4). LPE has smaller proportions of insertions with high divergence values from their consensus sequences. Either TE insertions, especially DNA transposon types, have occurred more recently than in *L. fuscus*, or older insertions have been lost from the LPE genome by deletions (consistent with presence of fewer sequences at all divergence levels than in *L. fuscus*; alternatively, as RepeatModeler analysis is not designed for data with the low coverage available from LPE, rare repeat types may be missed).

**Fig. 4:**
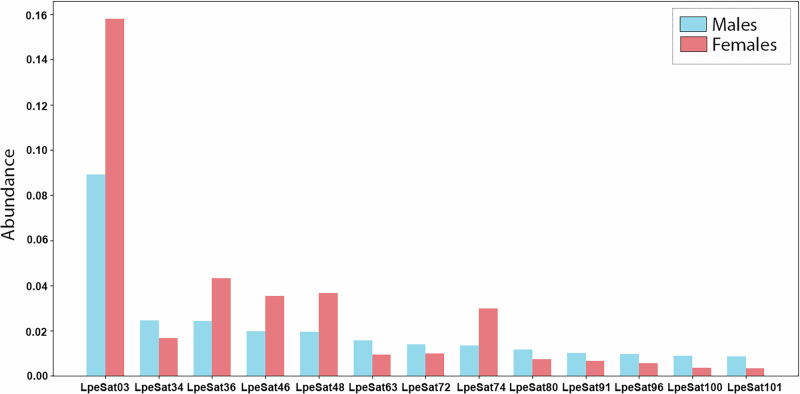
LpeSatDNAs that display the most significant differential abundances in males and females. The *Y*-axis shows the estimated abundance of each family named on the *X*-axis. Values square root-transformed (√*x*) to enhance visualization of smaller bars.

We next examined satellite DNAs, which can sometimes cause expansions of genome regions that recombine rarely, particularly pericentromeric regions, as found in *Drosophila* species^26^. In the nearly 1× coverage representative LPE genome sequences (see the “Methods' section), we identified 104 satDNA sequences, hereafter termed LpeSatDNAs, many of them A + T rich (range 21–83% AT, average 56%, as is frequently found). Most LpeSatDNA repeat units were >100 bp; their lengths range from 21 to 6930 bp (for LpeSat07 and LpeSat16, respectively), and the average was 429 bp. All 104 LpeSatDNAs were found in both sexes, but in varying abundances, of which the five with lower abundances were enriched in the female DNA sequence, and eight with low abundances were enriched in the male sequences (Fig. 4**;** Supplementary Fig. 6**;** Supplementary Table 3).

### In silico and in situ mapping of selected LpeSatDNAs against the *L. fuscus* genome

In the genome assembly of the distant outgroup *L. fuscus* (GCA_031893025.1), in silico mapping of LpeSatDNA sequences detected only four out of the 11 LpeSatDNAs mapping in LPE, indicating that, unsurprisingly, these repeat sequences are ephemeral and turn over between species within the genus, with only some sequences being preserved over the estimated ~28 MYA separating *L. pentadactylus* and *L. fuscus*. Among the four shared LpeSatDNAs, only LpeSat01-35 (by far the most abundant one in LPE) yielded positive hybridization signals in our in-silico mapping experiments, mostly in or near the terminal regions of all *L. fuscus* chromosomes (Supplementary Fig. 7).

### Mapping of LpeSatDNAs on LPE mitotic chromosomes

**Fig. 5:**
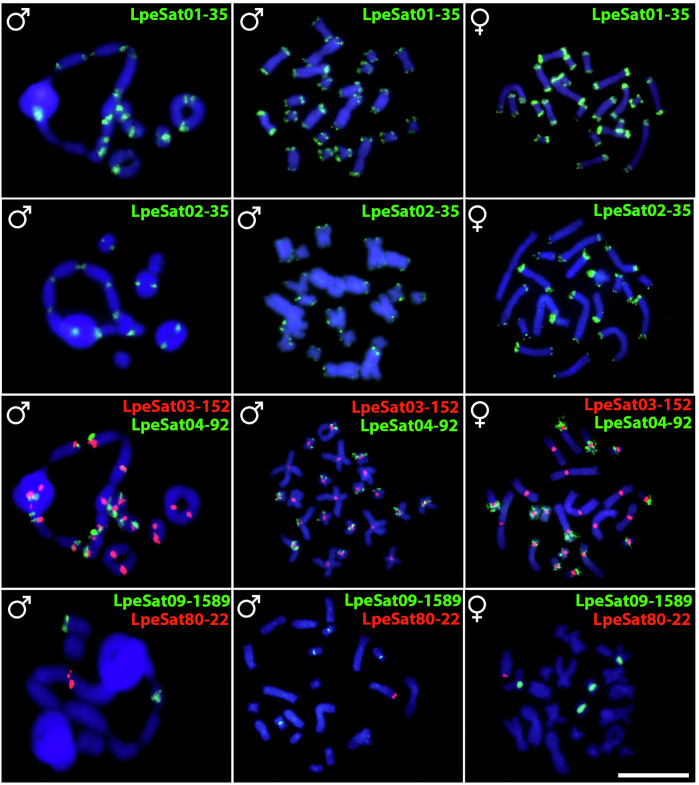
Fluorescence in situ hybridization in LPE using different LpeSatDNAs probes. Male meiotic cells of *L. pentadactylus* (first column) and mitotic metaphases of a male and female (second and third columns, respectively) after FISH with the LpeSatDNA families indicated; these were detected on both autosomes and sex chromosomes. Green and red indicate staining with Atto-488-dUTP and Atto-550-dUTP, respectively. Bar = 10 μm.

**Fig. 6:**
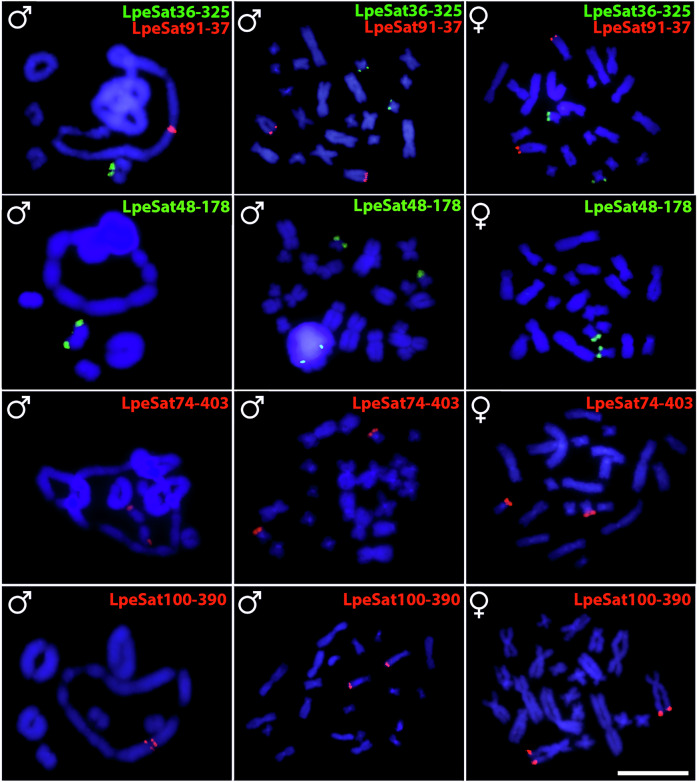
Fluorescence in situ hybridization in LPE using different LpeSatDNAs probes. Male meiotic cells of *L. pentadactyus* (first column) and mitotic metaphases of a male and female (second and third columns, respectively) after FISH with the LpeSatDNA monomers indicated; these were also detected on both autosomes and sex chromosomes. Green and red indicate staining with Atto-488-dUTP and Atto-550-dUTP, respectively. Bar = 10 μm.

Eleven of the 18 LpeSatDNAs for which primers were designed (see the “Methods” section) showed positive hybridization signals on mitotic chromosomes of both LPE males and females (Figs. 5 and 6**;** Table 2). LpeSat01 was again detected in terminal regions of all chromosomes, and LpeSat03 in centromere-proximal regions. LpeSat02 and LpeSat04 showed signals on almost all chromosomes at their terminal and centromere-proximal regions, respectively. We detected hybridization signals of LpeSat09 in the pericentromeric regions of two chromosome pairs within the ring multivalent. LpeSat36, LpeSat48, LpeSat74, LpeSat91, and LpeSat100 hybridized with a single pair of chromosomes, while LpeSat80 was present in a single homologue from pair 3 (Figs. 5 and 6).

**Table 2:** Chromosomal distribution of DNA repeats (satDNA and rDNA sequences) on the LPE and LPA chromosomes

	Numbers of chromosomes with hybridization sites	
DNA repeat	Locations in LPE	Locations in LPA
LpeSat01	All 10 chromosomes in the ring + 6 bivalents (t)	All 8 chromosomes in the ring + 7 bivalents (t)
LpeSat02	All 10 chromosomes in the ring + 4 bivalents (t)	4 chromosomes in the ring + 2 bivalents (t)
LpeSat03	All 10 chromosomes in the ring + 6 bivalents (c)	2 chromosomes in the ring + 1 bivalent (c)
LpeSat04	All 10 chromosomes in the ring + 2 bivalents (pc and t)	All 8 chromosomes in the ring + 7 bivalents (pc and t)
**LpeSat09**	2 chromosomes in the ring (**pair 7**) + 2 bivalents (pc and t)	No signal
LpeSat36	Only a single bivalent (t)	No signal
LpeSat48	Only a single bivalent (t)	No signal
LpeSat74	Only a single bivalent (t)	No signal
**LpeSat80**	Only 1 chromosome in the ring (one homologue of chromosome **pair 3**) (t)	No signal
**LpeSat91**	Only 2 chromosomes in the ring (**pair 4**) (t)	No signal
**LpeSat100**	Only 2 chromosomes in the ring (**pair 3**) (t)	No signal
18S rDNA	Only one bivalent (t)	Only one bivalent (t)
**5S rDNA**	Only 2 chromosomes in the ring (single homologues of each of chromosome **pairs 3 and 5**) (t)	Only one bivalent (t)

Bold font indicates the DNA repeats that identify individual chromosomes in the LPE decavalent meiotic ring. The regions where repeats were detected are indicated as follows: telomeric regions (t), centromeric regions (c), and pericentromeric regions (pc).

### Mapping of rDNAs, telomeric repeats, and comparative genomic hybridization (CGH) on LPE mitotic chromosomes

The telomeric probe (see the “Methods” section) mapped to the terminal region of all chromosomes in both species, identifying the telomeres mentioned in what follows, and no interstitial telomeric sites were detected (Supplementary Fig. 8). Only pair 8 of the 11 LPE mitotic chromosome pairs carries 18S rDNA sites (Fig. 3). Sequential hybridization using 18S rDNA, LpeSat09, LpeSat36, LpeSat48, and LpeSat74 probes established that these sequences all occur on this chromosome (Supplementary Fig. 9), In females, the 5S rDNA probe hybridized to both homologues of pair 5, while in males, it hybridized only in a single homologue of each of pairs 3 and 5 providing further evidence that one translocation involved these chromosomal pairs (Fig. 3). Other than this, intraspecific CGH experiments in LPE to search for sex-specific sequences revealed only overlapping signals in the centromeric regions, indicating similarity between the male and female genomes (Supplementary Fig. 10).

### Mapping of LpeSatDNAs on LPE and LPA meiotic chromosomes

**Fig. 7:**
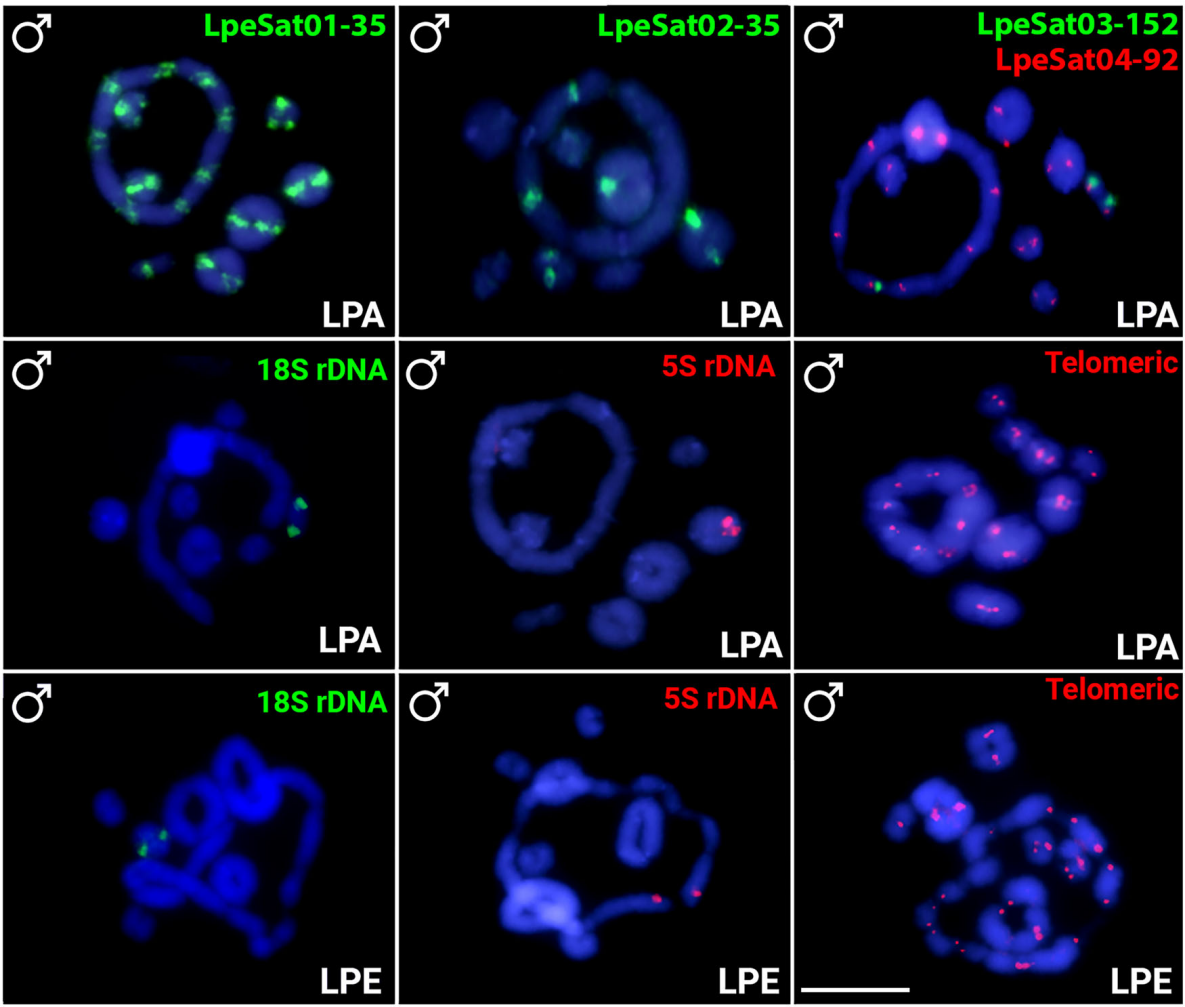
Repetitive DNAs hybridized in *L. pentadactylus* and *L. paraensis.* Male meiotic cells hybridized with LpeSatDNAs (LPA only) and rDNAs (both LPA and LPE). Green and red indicate staining with Atto-488-dUTP and Atto-550-dUTP, respectively. Scale bar = 10 μm.

FISH experiments revealed that 18S rDNA sites mapped to a single bivalent in meiotic preparations from both species, as does 5S rDNA in LPA, whereas, as just described, it is detected on chromosome 5 in females in the LPE ring of 10, or 3 and 5 in males (Fig. 7).

We found three main LpeSatDNA hybridization patterns present both on bivalents and on one or more chromosomes in the ring (LpeSat01, LpeSat02, LpeSat03, LpeSat04, and LpeSat09), 2) present only on bivalents (LpeSat36, LpeSat48, and LpeSat74), and 3) present exclusively on one or more chromosomes in the ring (LpeSat80, LpeSat91 and LpeSat100) (Table 2, Figs. 5 and 6). Two of the four most abundant LpeSatDNAs showed hybridization signals on all the chromosomes (Table 1), but LpeSat02 mapped only to three bivalents and LpeSat004 to just one (Fig. 7).

### Identifying the chromosomes in the LPE meiotic ring

**Fig. 8:**
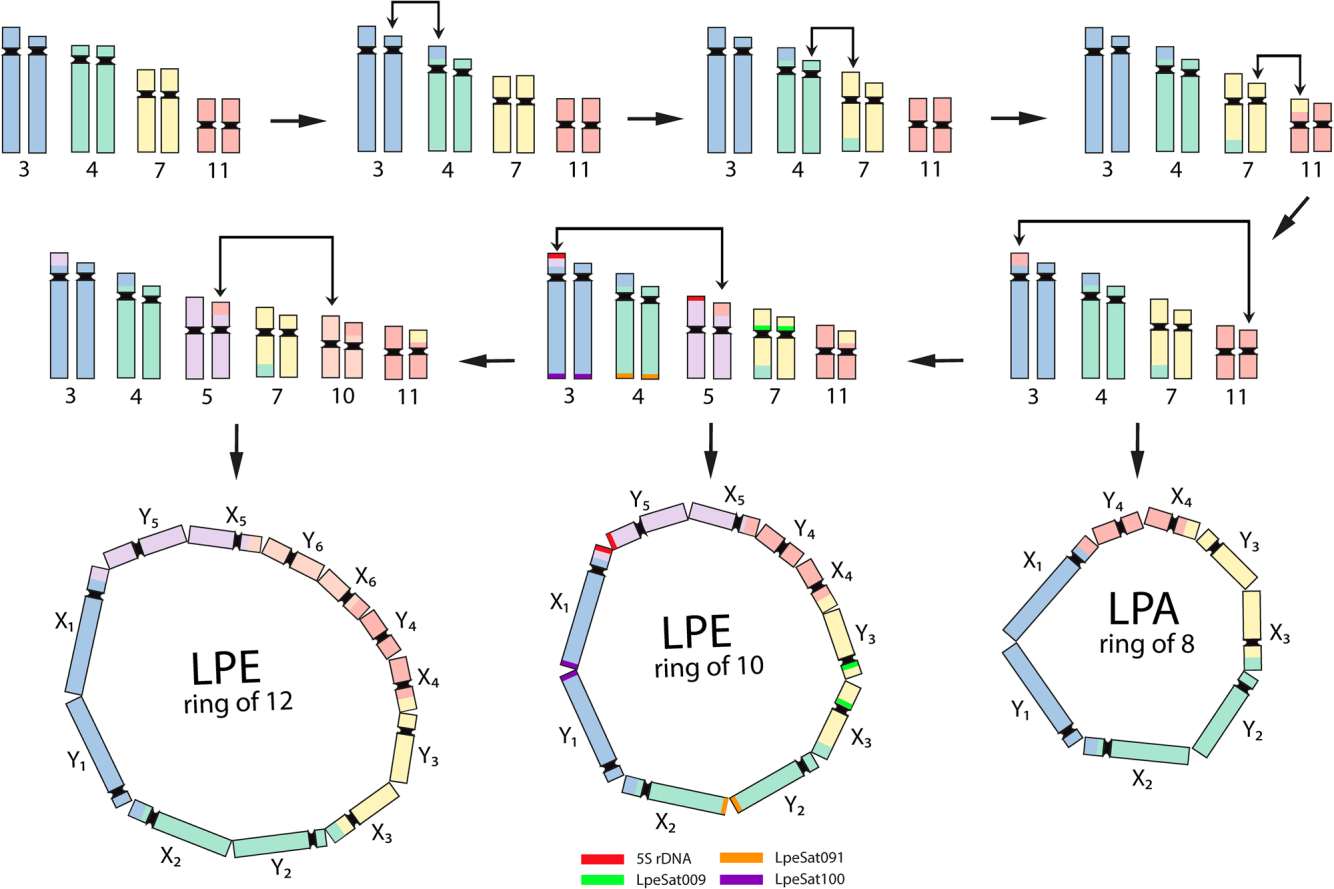
Schematic ideograms illustrating a working hypothesis for ring formation involving consecutive reciprocal translocations. The upper part shows the chromosome numbers identified as becoming parts of the rings, numbered according to the mitotic karyotype in Fig. 3a. Starting with a large ancestral XY sex chromosome pair (in this hypothesis, it is assumed to be the heteromorphic pair 3 (following ref. ^13^) and is therefore labelled X_1_ in the diagrams), successive translocations involving autosomes (symbolized by lines with arrows at both ends) resulted in the formation of rings of increasing size, eventually including eight chromosomes, as observed in LPA, and even more in LPE (10 and 12), as shown in the bottom row. The positions of repetitive DNA landmarks that distinguish the chromosomes in the LPE meiotic ring of 10 are also shown, and their correspondences with the numbers in the LPE mitotic karyotype in Fig. 1a are indicated by different colours. The black regions represent the centromeres of the chromosomes that are detected by the presence of heterochromatin (these are not shown to scale, as the amounts of DNA cannot be determined by cytological observation, as levels of condensation differ between heterochromatin and euchromatin).

Our in-situ mapping of SatDNAs to meiotic and mitotic chromosomes of LPE allowed us to identify four (pairs 3, 4, 5, and 7) of the five chromosomes involved in the ring of 10 (see Table 2 and Fig. 8, showing ideograms of the multivalents). Co-hybridization of LpeSat03-152 and LpeSat04-92 further showed that pairs 1 and 2 are not part of the decavalent and hybridization in mitotic chromosomes shows that neither of these chromosomes exhibits co-localization of these satDNAs in their centromeric regions (Fig. 5). Complementary meiotic investigations confirm that the two largest bivalents also lack this co-localization (Fig. 5). The same applies to pair 8, a small bivalent that carries the 18S rDNA loci in this species (and also in LPA; Fig. 7), confirming the previous inference in individuals with a ring of 12, whose chromosome pair carrying this locus was not in the multivalent^13^. Chromosomes 3, 4, and 7 are part of the ring of 10, as shown by the presence of LpeSat100-390 (on pair 3), LpeSat91-37 (pair 4), and LpeSat09-1589 (pair 7) within the multivalent (Figs. 5 and 6). We also established that pair 5 is in the LPE multivalent, based on the presence of the 5S rDNA locus on this chromosome in the decavalent; this indicates a rearrangement between pairs 3 and 5 (Figs. 3 and 7). The final component of the ring multivalent is the smallest chromosome, pair 11 (Fig. 3).

## Discussion and conclusions

Ring multivalents or chains must be initiated by a reciprocal translocation between two metacentric chromosomes, usually in centromere-proximal regions, and further such events can add more chromosomes to the system. The events described here are an example of the situation when the sex chromosome pairs are involved, forming neo-sex chromosomes, as in the monotreme mammals^12,16,17^. These are not sex chromosome turnovers, as the ancestral sex chromosome arms remain as part of the system, and, as far as is known, the sex-determining system is not changed or moved to a different genomic location, as occurs in turnovers in other systems (reviewed in ref. ^35^).

Figure 8 shows schematic ideograms of possible steps involved in the formation of the *Leptodactylus* rings. None of the chromosomes within the multivalent could be definitively identified in the other ring-bearing species (LPA) based on our in-situ mapping results (the species are clearly distantly enough related that their satellites have turned over, consistent with their not being very close relatives, see Fig. 2 above), and mitotic preparations of sufficient quality to detect heteromorphism are lacking for this species. However, the strong conservation of chromosome sizes across *Leptodactylus* species (see above), including between LPE and *L. fuscus* (Supplementary Table 1 and Supplementary Fig. 2), suggests that four elements in the LPA ring are shared by both species’ multivalents. First, the great inequality of the arm lengths identifies chromosome 4 in both LPE and *L. fuscus*, and a chromosome with this morphology is visible in the male meiotic rings of both species, supporting the conclusion that it is one component of both species’ rings. Second, one of the two other largest chromosomes in the LPA ring is probably pair 3, as this is heteromorphic in an LPE population with a ring of 12 chromosomes, and may correspond to an ancestral XY pair^13^. It would then follow that the other large chromosome in the LPA male meiotic ring must be smaller than pair 3 (and 4), and since the sizes of pairs 5 and 6 are very similar to that of pair 4, it probably corresponds to pair 7, which is considerably smaller. As the small element in the LPA ring is considerably smaller than pair 7, it probably corresponds to pair 11. Based on similar evidence, Fig. 8 shows chromosomes 5 and 10 as having been added in the LPE lineage since the split from LPA, implying that one member of LPE chromosome pair 5 expanded since becoming Y-linked. These proposed chromosome homologies can be tested once genes can be assigned to these chromosomes by long-read sequencing, assembly and annotation, as has been possible in monotremes^18,36^.

In the genus *Leptodactylus*, only the two species studied here have so far been shown to have systems with multiple sex chromosomes, but most species remain unstudied, and more with rings may exist, and meiotic investigations of more species are needed. Our inferred phylogeny does not support a common origin for these meiotic rings (Fig. 2). Sequence divergence estimates are needed to test this and understand when the rearrangements occurred and whether they occurred at a high rate. Below, we discuss the hypothesis that hybridization can help explain the occurrence of multiple rearrangements in a short evolutionary time.

The Introduction outlined factors that allowed such rearrangements, and we next briefly discuss the relevance of these ideas to neo-sex chromosome evolution, including what consequences this may have had and how these effects may help to understand how the rings evolved and shed light on Y chromosome degeneration.

### Pre-conditions allowing translocations to establish if they occur

Our results confirm previous observations that LPE male meiosis has properties facilitating translocations by minimizing production of unbalanced gametes, as reviewed by ref. ^37^. They have asynaptic regions in the centres of the chromosomes in the ring during anaphase I of male meiosis, and terminal chiasma localization^13^. Furthermore, adjacent centromeres in the multivalent appear to segregate in an alternating pattern in male meiosis^31^. Noronha^19^ also investigated meiotic cells of LPE males with a ring of 10, using immunofluorescence to analyse segregation and the possible effects of the dodecavalent on fertility, and found no reduction in the fertility of carriers of individual rearrangements.

Genetic maps have confirmed strong terminal crossover localization not specific to the sex chromosome pair, but specific to male meiosis, in >30 species with male heterogamety in the families Ranidae, Hylidae, and Pipidae (reviewed in refs. ^38,39^). Species with female heterogamety may differ in the same way between male and female meiosis, based on observations of chiasmata in lampbrush chromosomes in female meiosis of the Kajika frog *Buergeria buergeri*^40^. The terminal pairing observed in *Leptodactylus* (with male heterogamety), and crossover localization not specific to the sex chromosome pair, may therefore reflect an ancestral genome-wide pattern in male meiosis. One reason that these species have translocation rings is thus probably simply that the deleterious effects of the translocations are minor.

### Possible genomic associations with translocation breakpoints

Analyses in several species have suggested the possibility that satellite sequences can cause rearrangements such as the translocations studied here^41–44^, and some studies have detected satDNAs in fusion or fission breakpoint regions^43,45–47^. This is plausible, as tandem repeats can induce ectopic recombination^48^. For example, in house mice and humans, distinct chromosomes containing the same satDNA family have experienced Robertsonian translocations^49^, though this is not evidence that the satDNAs are involved unexpectedly often. They may simply be abundant in repeat-rich regions that recombine rarely, such as pericentromeric regions, as outlined in the Introduction. It therefore remains unclear whether the satDNA sequences analysed here are associated with the evolution of the multiple rearrangements that created the multivalents. In LPE, most LpeSatDNAs are found near the ends of chromosomes (Figs. 5 and 6), whereas the reciprocal translocations probably involved rearrangements with breakpoints in the middle regions, so that adjacent centromeres segregate in an alternating manner. This restricts the candidates for involvement of satellites to the few that are found in the middle regions (e.g., LpeSat03 and LpeSat09).

TEs also cause genomic rearrangements, including deletions, insertions and translocations^50,51^. Large recent expansions of TEs might have preceded the rearrangements^52–54^. For instance, DNA transposon EnSpm elements appear to be responsible for repeated translocations of sex-determining regions in various *Takifugu* (pufferfish) species^55^. Our first step towards asking whether TEs could have been involved in the rearrangements that formed the rings found in some *Leptodactylus* species (either promoting the rearrangements, or accumulating as a consequence of them), revealed somewhat smaller genome-wide TE abundance, and does not suggest recent expansion of TEs in the genomes of the ring-forming species as a whole, compared with *L. fuscus*, which does not have multiple sex chromosomes (however, this species diverged long before the rings appear to have evolved in LPE and LPA, which post-dates the split with *L. labyrinthicus*, that also lacks multiple sex chromosomes, and is probably much more closely related (see Fig. 2).

Specific TE types may have expanded, but such expansions occur in most species that have been studied, including in *L. fuscus*. Our currently unassembled genome sequences leave it unclear whether TE accumulation (or accumulation of any specific TE types) occurred disproportionately in the breakpoint regions. Deeper sequencing and analyses of reliable LPE and LPA genome assemblies based on long reads can potentially answer these questions, along with manual curation of unknown TEs to help identify whether the genomes of lineages with rings carry genuinely lineage-specific TEs. Even with phased genomic sequence data to identify translocation breakpoints in multiple such species with suitable outgroups, however, it will be difficult to determine whether any TE-rich regions were the cause of the rearrangements, rather than reflecting accumulation after them.

### Effects of translocations on the evolution of the chromosomes involved

The ancestral pericentromeric regions in which the breakpoints probably occurred were presumably not already completely non-recombining, so the translocations may have created new regions in which recombination was reduced. These regions should then start accumulating repeats^53–57^, including both satellites and transposable elements. In species with heteromorphic sex chromosomes, repetitive DNA sequences are often highly enriched on, or even, in some cases, limited to, one or both sex chromosome(s) (e.g., refs. ^54, 56, 57^). However, although several LpeSatDNAs are more abundant in one sex than the other (Supplementary Table 3; Supplementary Fig. 6; Fig. 4), none show complete X- or Y-specificity. Other species with multiple sex chromosomes also exhibit minor or no differential accumulation of SatDNA sequences or heterochromatin in the multivalents^58–61^. Occurrence of satellite sequences on several different chromosomes and abundance differences among sampled individuals will also obscure sexual dimorphism.

If accumulation does occur in newly Y-linked regions, these regions should increase in size, while their X-linked counterparts should not, or should do so to a smaller extent. The cytogenetic results described here indicate expansion of one member of each pair of the ring chromosomes only for chromosomes 3 and 5 in LPE. The observation that most chromosomes in the two ring-forming frog species studied here have not become evidently heterochromatic (Fig. 3), suggests that none of the chromosomes in the ring carries large new non-recombining regions, or that these rings are too young for cytologically detectable expansions, or build-up of repetitive sequences detectable by staining for heterochromatin, to have occurred in most of them (though repeat accumulation in Y-linked regions is expected to occur rapidly after recombination is suppressed^52^). However, it seems unlikely that all the neo-sex chromosomes of these species are extremely young, since fixation of multiple rearrangements in the male genome of a species probably requires a considerable evolutionary time, even in a small population in which genetic drift allows rearrangements to increase in frequency^28^. The lack of a large build-up of heterochromatin and repetitive DNAs might, however, be explained by natural selection against changes causing size differences that impede correct segregation in a ring system, increasing the frequency of deleterious unbalanced gametes^2,62–64^. Sequence data therefore have better potential to test whether recombination has completely stopped, or whether crossover events occasionally occur in the neo-sex chromosome arms. Specifically, Y–X divergence estimates for the different Y–X pairs can test this and reveal when different genome regions stopped recombining.

### How old are the multivalents, and could they originate rapidly?

The finding that the LPE ring of 12 appears to share all four chromosomes that are present in the LPA ring (Fig. 8) suggests that both these species with multivalents probably split from a common ancestor that had already evolved such a ring, similarly to the situation in the monotremes, in which the platypus and echidna share most components of their rings, (see refs. ^16,17^). However, our preliminary data on sequence divergence between these two frog species (Fig. 2), and from outgroup species that lack them, suggest that ring systems might have evolved independently in LPA and LPE; however, the bootstrap support is weak for the topology clustering non-multivalent species *L. labyrithicus* with LPE, and we therefore cannot currently definitively assign the evolution of the multivalents to specific branches of the tree. However, the very small sequence divergence between LPA and *L. vastus* suggests that they should probably share similar rings, placing their origination at an unknown past time. If a multivalent system pre-dates the split of the lineages that include LPE and LPA, as suggested by the morphologies of chromosomes in the ring, we can use the closely related *L. vastus* as a proxy for LPA; the estimated time of the split from LPE is 26 million years ago, based on nine and three nuclear and mitochondrial genes, respectively^65^, and a molecular clock that yielded estimates broadly consistent with fossil ages^66–69^. As these frogs have 1–2 generations per year^70^, the rings could have evolved more than this number of generations ago. They could thus be of comparable age to those in the monotremes, which date back at least 55 MY, and the generation times are longer^18,36^. If ring systems evolved independently in the two frog lineages, the multivalents could be more recent, making this genus better suited than the monotremes for studying the details of how these systems evolved.

If recombination has completely stopped and sufficient time has passed since they did so, these regions should undergo genetic degeneration like that of other Y-linked regions, again making the loss of recombination potentially detectable. In addition to providing better and more direct estimates of the times at which different chromosomal elements in the rings in these frogs appeared, such sequence data would also provide estimates of the extent to which the different neo-Y-linked regions have lost genes, for comparisons with the monotremes (in which, Y–X sequence divergence is extreme^18^ and genetic degeneration is profound, with the neo-Y-linked regions retaining only very small proportions of the genes carried in the neo-X-linked regions). Such data on younger systems are needed, as the time course of Y chromosome degeneration is still very unclear.

Finally, we note that hybridization could potentially cause multiple rearrangements without a very long evolutionary time. This appears to be the case in the common shrew^71^ and the black muntjac, *Muntiacus crinifrons*^72,73^, although the exact mechanisms involved in the evolution of these multiple rearrangements remain unclear. If two populations’ chromosomes have undergone different translocations, their hybrids will be heterozygous for different rearrangements. The process is similar to that resulting in chromosomal polymorphisms within hybrid populations, specifically inversions in stickleback fish^74,75^ and frogs^76,77^. Recurring reciprocal translocations can produce substantial chromosomal chains in hybrid populations^77–81^, as documented in plant species with large multivalents unrelated to sex chromosomes^82^. Although hybridization has not been documented in *Leptodactylus*, it is plausible, as LPE and LPA are found in sympatry, and hybridization has been documented between other frog species^83–85^. The conserved chromosome number in *Leptodactylus* would probably allow correct meiotic pairing and segregation in hybrids. However, elements of the ring that are shared by both species probably pre-date the split of the species.

## Methods

### Samples, chromosomal preparation, DNA extraction, and C-banding

Table 1 lists the species used in this study, and the sampling locations (Fig. 1 shows a map of the sampling sites). Individuals were identified based on species-specific morphological traits and deposited in the frog collection of the Federal Institute of Education, Science, and Technology of the State of Pará (IFPa) and in the Federal University of São Carlos (UFSCar). The specimens were collected from wild populations with authorization from the Chico Mendes Institute for Biodiversity Conservation (ICMBIO) and the System of Authorization and Information about Biodiversity (SISBIO) under license number 96067-3. The experiments followed the ethical standards set by the Federal University of São Carlos Ethics Committee on Animal Experimentation (CEUA) under process number 7994170423. We have complied with all relevant ethical regulations for animal use.

Mitotic chromosomes were obtained from bone marrow of all six species in Table 1, except for LPA, following the protocol of ref. ^86^, and meiotic chromosomes were obtained from males following ref. ^87^. Genomic DNA (gDNA) of LPE males and females was extracted using the phenol-chloroform method ref. ^88^. C-positive heterochromatin was detected using C-banding^89^.

### Measurements of chromosome lengths

Images of mitotic metaphase from *L. fuscus* and *L. pentadactylus* were captured using an Olympus BX50 microscope (Olympus Corporation, Shikawa, Japan)with a total of 10 high-quality photographs for 5 male metaphases from each species, from which the lengths of the *p* and *q* chromosomal arms were measured. Chromosomes were numbered in descending order of size, after which centromere positions were assigned based on clearly observable constrictions. Lengths were measured using ImageJ software (version 1.53i)^90^, employing a uniform scale bar of 20 µm for all pictures. Median values for each chromosome were computed according to the methods outlined by ref. ^91^.

### Phylogenetic analysis

To investigate the phylogenetic relationship between the species studied, partial sequences 16S ribosomal DNA genes from selected *Leptodactylus* species (including the five species cytogenetically analysed in the present study) were obtained by polymerase chain reaction (PCR) using the universal primers 16Sar (5’-CGC CTG TTT ATC AAA AAC AT-3’) and 16Sbr (5’-CCG GTC TGA ACT CAG ATC ACGT-3’) described by ref. ^92^. The PCR procedure used a master mix including 5× buffer, dNTP mix, MgCl₂, 1.0 µL of each primer, Taq DNA polymerase, template DNA, and ultrapure water. The thermocycling conditions included initial denaturation at 94 °C for 5 min, followed by 30 cycles of denaturation at 94 °C for 45 s, annealing at 48.2–51.7 °C for 1 min and 30 s, and extension at 72 °C for 1 min and 30 s, with a final extension at 72 °C for 1 min. The PCR products were then sequenced with the ABI 3730 DNA Analyser (ThermoFisher Scientific). We included 16S sequences available in NCBI from two additional *Leptodactylus* species, *L. myersi* and *L. vastus* (KM091601.1 and MH004305.1, respectively). As an outgroup to root the trees, we used *Engystomops petersi* (NCBI code: EF011554.1). All sequences were aligned using MUSCLE with default parameters in MEGA v12 software^93^. The resulting alignments were manually inspected and trimmed to a uniform length of 470 base pairs. A model selection test was conducted under the maximum likelihood (ML) statistical framework, using the Complete Deletion option. Phylogenetic reconstructions were performed using the Maximum Likelihood method with 1000 bootstrap replicates, and the General time reversible (GTR) model was selected as the best-fit model according to the model test results. Rate variation among sites was modelled using a gamma distribution (G), in the option for non-coding sequence, and the nearest-neighbour interchange (NNI) heuristic method was used for tree inference. A matrix of pairwise divergence values is shown in Supplementary Table 4. Final trees were visualized and edited using IQtree (FigTree v1.4.4)^94^. 16S rDNA sequences from *L. pentadactylus, L. mystacinus, L. latrans, L. labyrinthicus, L. fuscus* and *L. paraensis* are deposited in the NCBI database under accession numbers PX048541, PX048542, PX048543, PX048544, PX048545, PX048546, respectively.

### Genome sequencing of *L. pentadactylus*

The genomic DNAs of one LPE male and one female were sequenced on the BGISEQ-500 platform at BGI (BGI Shenzhen Corporation, Shenzhen, China) (PE-150) and yielded total sizes of 2.46 and 2.43 Gb, respectively, representing 1× coverage. The raw reads are available on the Sequence Read Archive (SRA-NCBI) under accession numbers SRR30896012 (male) and SRR30896011 (female). Satellite DNA (satDNA) sequences characterized from LPE individuals (see below) are deposited in the GenBank database under accession numbers (PQ462747–PQ462850).

### Preliminary repetitive DNA analyses in *L. fuscus* (LFU) and *L. pentadactylus* (LPE)

Currently, the only species in the genus with a chromosome-level genome assembly is *L. fuscus* (2*n* = 22), based on long reads of a male individual collected in Bogotá (Colombia) (GCA_031893025.1). Its estimated haploid genome size is 2.3 Gb, and it does not have a ring sex chromosome system. No genome assembly is available for either of the ring-forming *Leptodactylus* species. As mentioned above, *L. fuscus* is estimated to have diverged from LPE about 27 MYA. As genome and chromosome-wide synteny is very well conserved within frogs, even across highly diverged (~250 MYA) groups^34^, we used the *L. fuscus* assembly to map LpeSatDNAs and infer the chromosomal distributions of repeats.

As a preliminary evaluation of repetitive sequence content in LPE (with no genome assembly yet available) and comparison with that of *L. fuscus*, we first ran RepeatModeler2^95^ on the *L*. *fuscus* assembly with the -LTRStruc option enabled to include repeats ascertained based on their structures as well as their sequences. The resulting de novo transposable element (TE) library included sequences ranging from 27 to 13,275 bp. To quantify the repetitive fraction of the *L. fuscus* genome and the transposable elements in trimmed short-read data from LPE **(**Supplementary Fig. 11), this library was used as input for RepeatMasker^96^. As a rough indicator of the expansion history of the TE types, we used “repeat Landscape” analysis. This estimates Kimura 2-parameter (K2P) divergence values for each transposable element (TE) from the inferred consensus sequence of its type. To do this, we used the calcDivergenceFromAlign.pl script in the RepeatMasker package^96^. To more accurately assess TE abundance in LPE males and females, we also used DNApipeTE, which is optimized for the classification and annotation of repetitive DNAs in low-coverage data (<1×) without requiring a genome assembly^97^.

### Satellitome analysis

The LPE satDNAs were identified following the pipeline described in ref. ^98^ together with analysis with tools in the Tandem Repeat Analysis (TAREAN) package^99,100^ available at https://galaxy-elixir.cerit-sc.cz/. First, low-quality reads were filtered using Trimmomatic^101^, with the following parameters: LEADING:3 TRAILING:3 SLIDINGWINDOW:4:20 MINLEN: 100 CROP: 101. Then 2 × 500,000 reads were randomly selected using SeqTK (https://github.com/lh3/seqtk) and analysed with TAREAN. Candidate satDNA sequences identified in TAREAN were filtered from the raw reads using the DeconSeq tool^102^, and this process was repeated until no new candidates were found. To avoid multigene family sequences identified by TAREAN, the candidate satDNAs were aligned against multigene families with GENIOUS 6.1.8 (Biomatters) and any sequences with similarity to multigene families were removed. To prevent redundancy within the set of satDNAs, we used a crossmatch search in RepeatMasker^96^ and aligned each satDNA against the entire set of satDNAs. Genomic abundances were estimated by randomly selecting 10,000,000 raw reads and aligned to the set of satDNAs using a RepeatMasker script (https://github.com/fjruizruano/satminer/blob/master/repeat_masker_run_big.py) Divergence values for each member of each satDNA family from the family’s inferred consensus sequence were calculated for all site types using the calcDivergenceFromAlign.pl script^98^ and the proportions of sequences with different abundances were plotted against the divergence values using the Kimura 2-Parameter correction for saturation (repeat landscape plots). The satDNA families were named in order of decreasing abundance in the genome, following ref. ^98^.

### Polymerase chain reaction amplification of LpeSatDNAs

We designed primers for 18 LpeSatDNAs (Supplementary Table 5) for in situ mapping and amplified them using polymerase chain reaction (PCR). From the 10 most abundant LpeSatDNAs, we excluded LpeSat007 due to its short motif length (21 bp). We also included 9 other sequences that, whether they were abundant or not, showed male-to-female (M/F) or female-to-male (F/M) abundance ratios >1.4 and differed significantly from 1 (STATs), suggesting a sex difference in abundance (Supplementary Table 3). The PCR protocol included an initial denaturation step at 95 °C for 5 min, followed by 32 cycles with 95 °C for 40 s, 60 °C for 40 s, 72 °C for 50 s, and a final extension at 72 °C for 5 min. We confirmed amplification of the LpeSatDNAs by electrophoresis on 2% agarose gels and measured the concentration of the PCR products using a NanoDrop Spectrophotometer (ThermoFisher Scientific, Branchburg, NJ, USA).

### Probe preparation and fluorescence in situ hybridization (FISH)

The 5S and 18S rDNA probes were obtained via PCR, following refs. ^103, 104^, respectively. Telomeric sequences (TTAGGG)n were generated by PCR using the (TTAGG) and (CCTAA) primers described by ref. ^105^ in the absence of a DNA template.

All the aforementioned repetitive DNAs (i.e., rDNAs, telomeric, and the 14 amplified LpeSatDNA sequences) were labelled using the nick translation kit from Jena Bioscience (Jena, Germany), incorporating the fluorophores Atto488-dUTP (green) or Atto550-dUTP (red) according to the manufacturer’s instructions. Five satDNAs with motifs smaller than 40 bp (named LpeSat001, LpeSat002, LpeSat080, LpeSat091, and LpeSat096) were indirectly labelled with Biotin and further detected using Streptavidin-FITC (Sigma).

FISH experiments were performed under high-stringency conditions^106^. Each hybridization mix was composed of 200 ng of labelled probe plus 50% formamide, 2 × SSC, 10% SDS, 10% dextran sulfate, and Denhardt’s buffer at pH 7.0 in a total volume of 20 µl. The slides were dehydrated in ethanol (70%, 85%, and 100%) before counterstaining chromosomes with DAPI, mounted in Vectashield (Vector Laboratories, Burlingame, USA).

### Comparative genomic hybridization (CGH)

CGH experiments were conducted to examine genetic differentiation between males and females of LPE. The gDNAs of males and females were labelled with Atto550-dUTP (red) and Atto488-dUTP (green), respectively, using the nick-translation protocol (Jena Biosciences, Jena, Germany) and further hybridized against the male mitotic and meiotic chromosomes. To block the common repetitive sequences, we used unlabelled C0t-1 DNA (gDNA enriched in highly and moderately repetitive sequences) produced according to ref. ^107^. The hybridization mix was composed of 10 µg of unlabelled female-derived Cot-1 DNA and 500 ng of both labelled male and female gDNAs. After using ethanol-precipitation, the pellet was air-dried and well mixed with 20 μL of hybridization buffer (Denhardt’s buffer, pH 7.0), composed of 50% formamide, 2% 2 × SSC, 10% SDS, and 10% dextran sulfate. The CGH experiments followed the methodology detailed in ref. ^108^.

### Statistics and reproducibility

To confirm the 2*n* number, karyotype structure, and FISH results, at least 30 metaphase spreads per individual were examined. Images were captured with CoolSNAP on an Axioplan II microscope (Carl Zeiss Jena GmbH, Germany) and processed with ISIS (MetaSystems Hard & Software GmbH, Altlussheim, Germany). The map was generated using QGIS 3.32 (Lima) with a Natural Earth package and riverine information from HydroRIVERS140.

### Reporting summary

Further information on research design is available in the Nature Portfolio Reporting Summary linked to this article.

## Supplementary information

Supplementary Information

Description of Additional Supplementary Materials

Supplementary Data

Reporting Summary

## Data Availability

Sequencing data that support the findings of this study have been deposited in the Sequence Read Archive (SRA-NCBI) under accession numbers SRR30896012 (male) and SRR30896011 (female). Satellites DNA (satDNA) sequences characterized from *Leptodactylus pentadactylus* individuals are deposited in the GenBank database under accession numbers (PQ462747–PQ462850). 16S rDNA sequences from *L. pentadactylus, L. mystacinus, L. latrans, L. labyrinthicus, L. fuscus* and *L. paraensis* under access numbers PX048541, PX048542, PX048543, PX048544, PX048545, PX048546, respectively (https://www.ncbi.nlm.nih.gov/). The source data for Fig. 4 can be found in the supplementary Data.
