# Supplementary material for: Investigating the evolution of large meiotic rings of multiple X and Y sex chromosomes in two Leptodactylus frog species (Anura, Leptodactylidae)
Source: Commun Biol. 2025 Nov 21;8:1636. doi: 10.1038/s42003-025-09151-z (PMC12638755; doi:10.1038/s42003-025-09151-z)
Supplement: Supplementary file 1 — Supplementary Information [file 42003_2025_9151_MOESM1_ESM.docx]

**Supplementary Material**

**
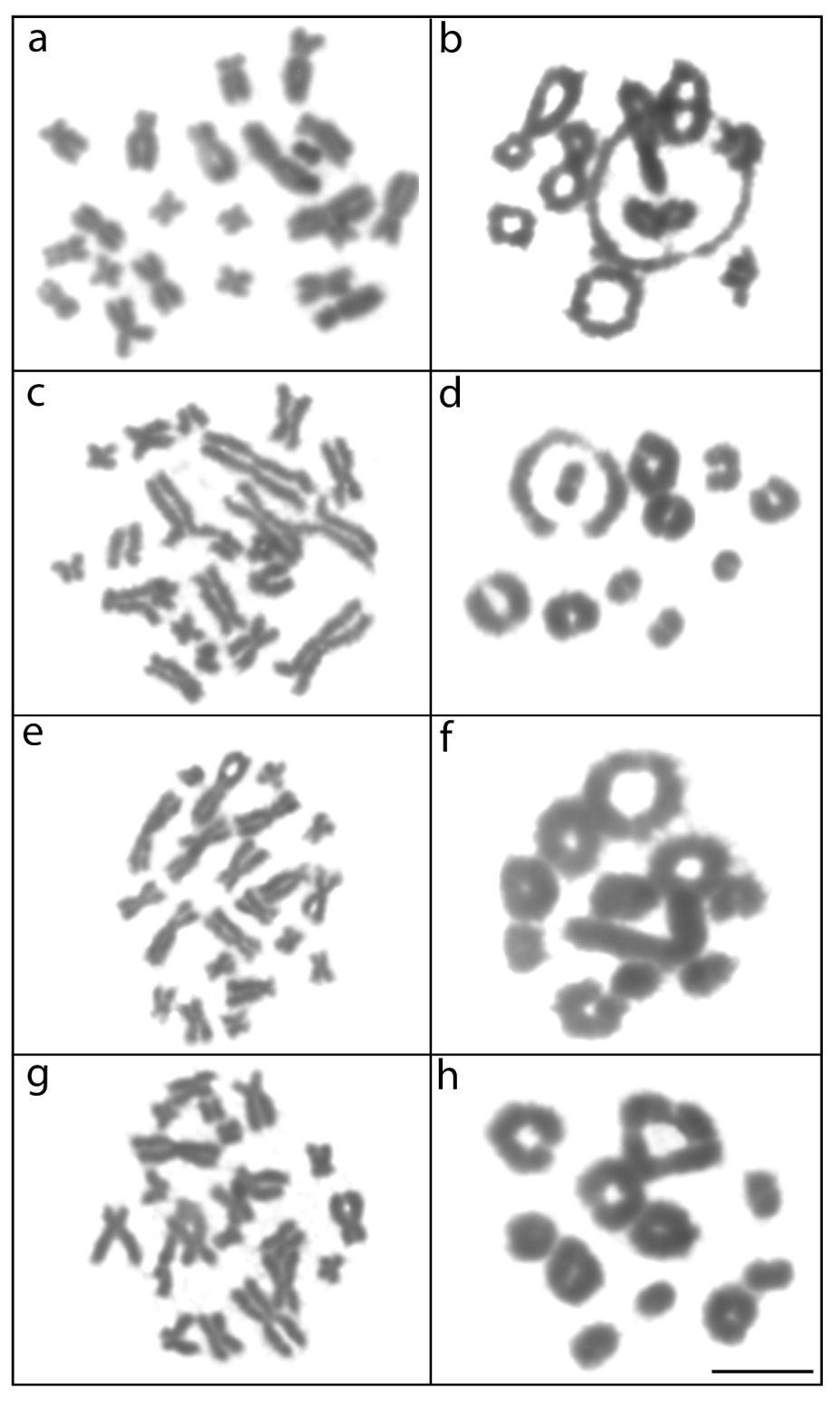
**

**Supplementary Figure 1:** Mitotic metaphases (a, c, e, and g) and meiotic metaphase I (b, d, f, and h) from *L. fuscus* (a and b), *L. mystacinus* (c and d), *L. latrans* (e and f), and *L. labyrinthicus* (g and h). Bar = 10 μm.


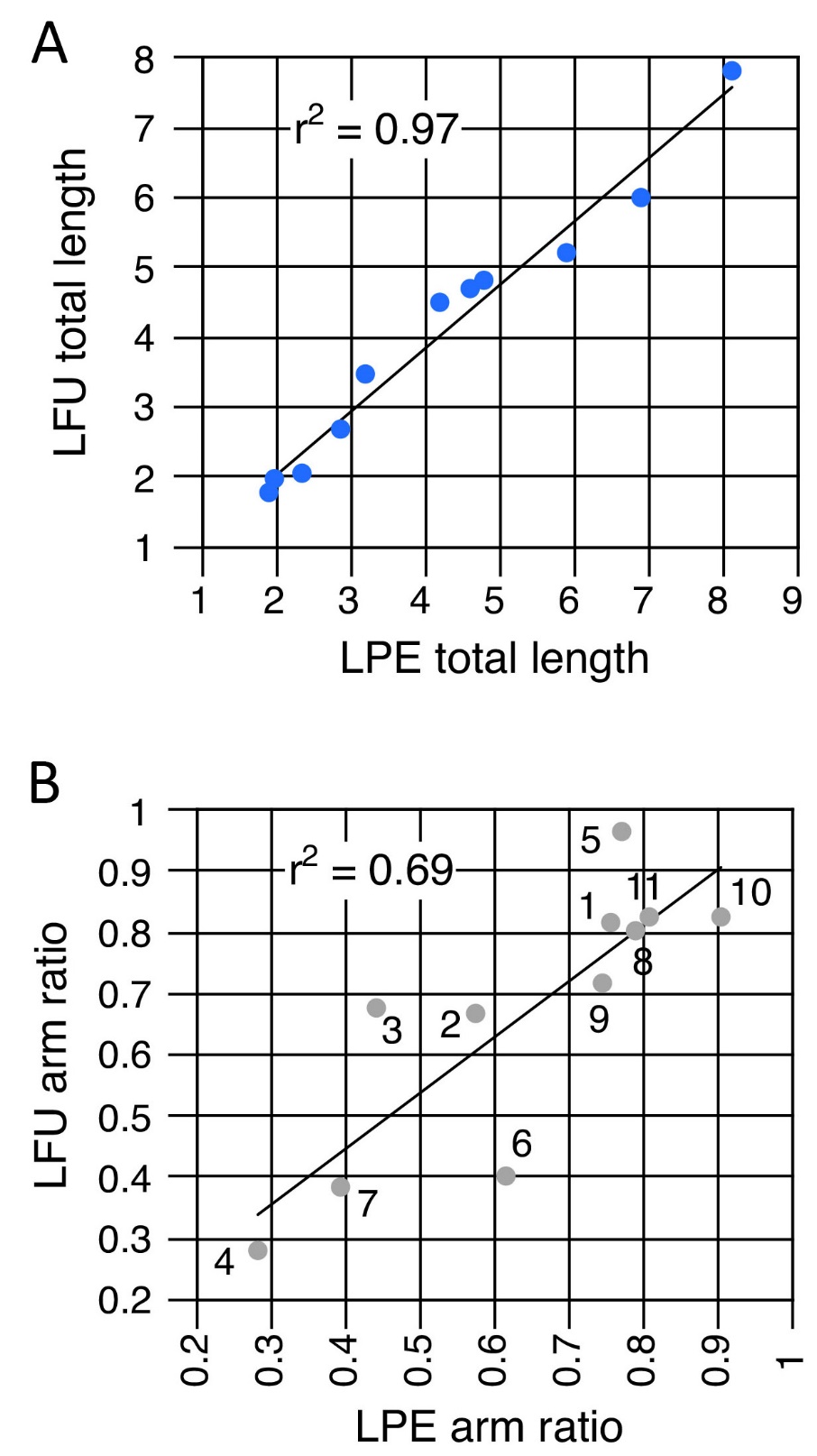


**Supplementary Figure 2.** Linear regression between the total chromosome lengths (A) and arm rations (B) of *L. fuscus* (LFU) and *L. pentadactylus* (LPE). The solid line represents the best-fit linear regression. The coefficient of determination (r²) indicates the degree of correlation between chromosome sizes in both species, suggesting a strong relationship between the measurements.

**
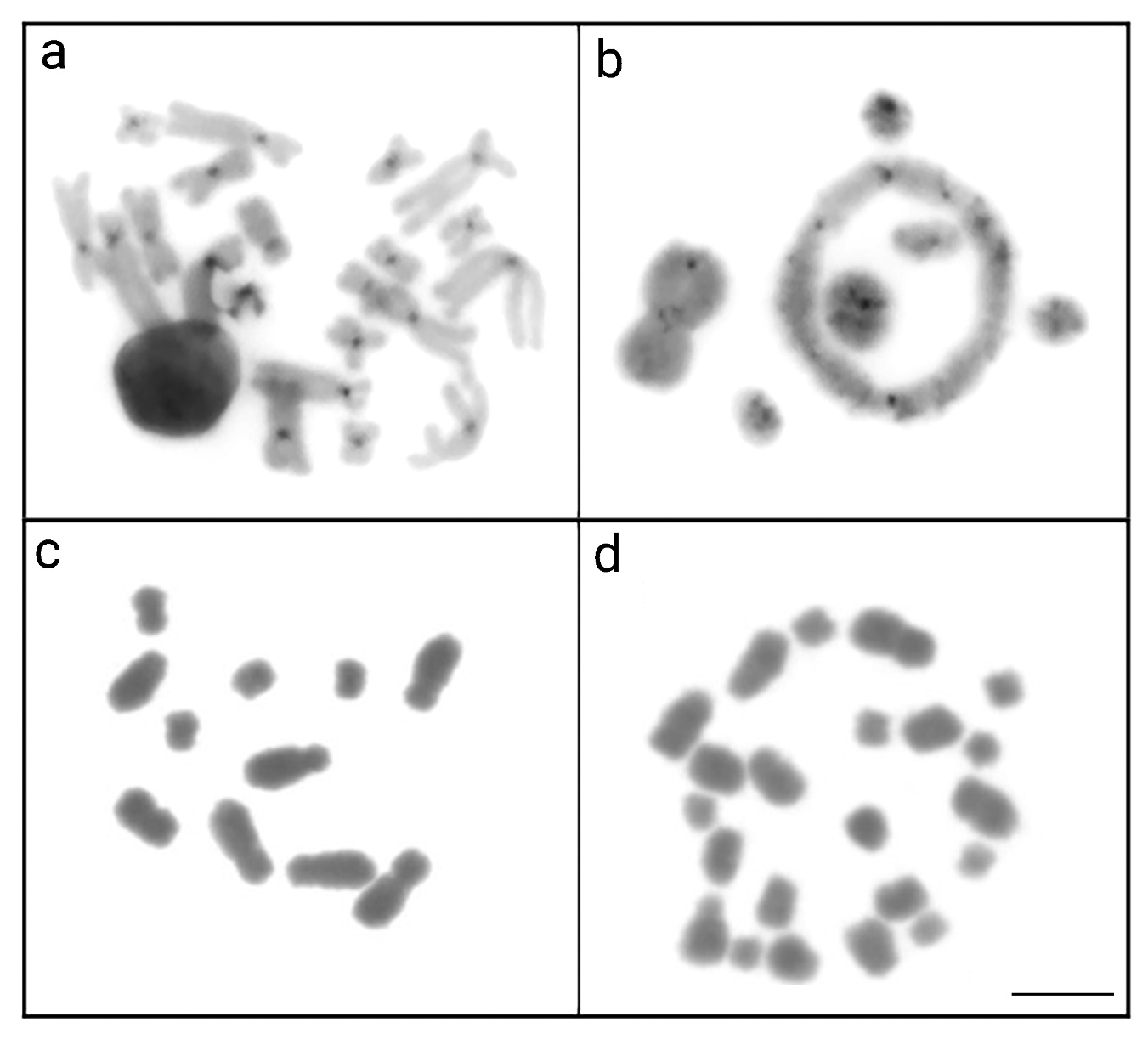
**

**Supplementary Figure 3.** Mitotic **(a)** and meiotic preparations **(b, c, d)** from *Leptodactylus paraensis* (LPA). a and b highlight the C-positive heterochromatin detected in the pericentromeric regions of all chromosomes. **c.** Metaphase meiotic II preparation with 11 chromosomes. **d** Metaphase meiotic I preparation with 22 chromosomes. Bar = 10 μm.


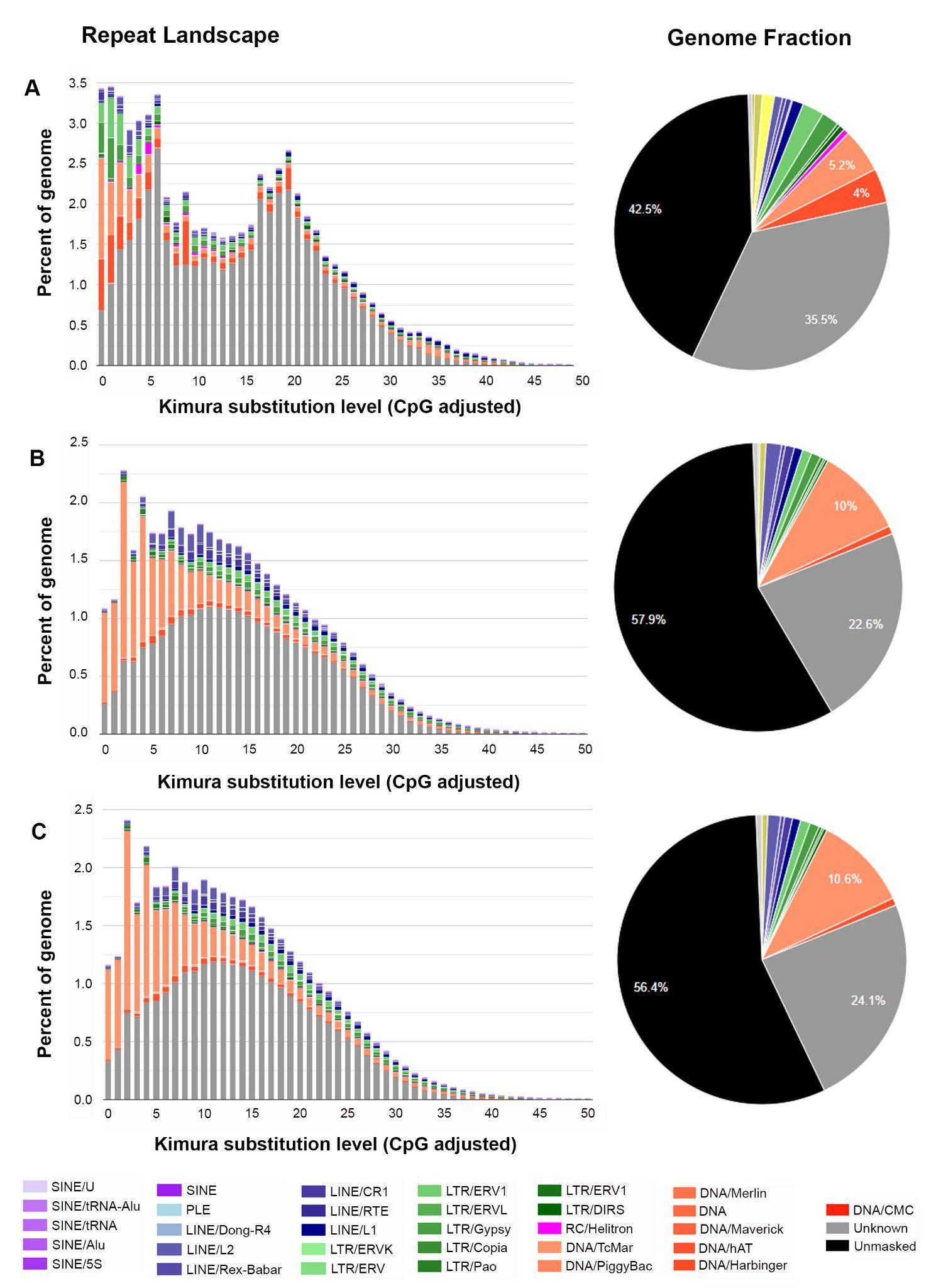


**Supplementary Figure 4**: Repeat landscape and pie chart of repetitive DNA class from *Leptodactylus fuscus* (A), *Leptodactylus pentadactylus* female (B) and male (C) using RepeatModeler and RepeatMasker as described in **Supplementary figure 11**.


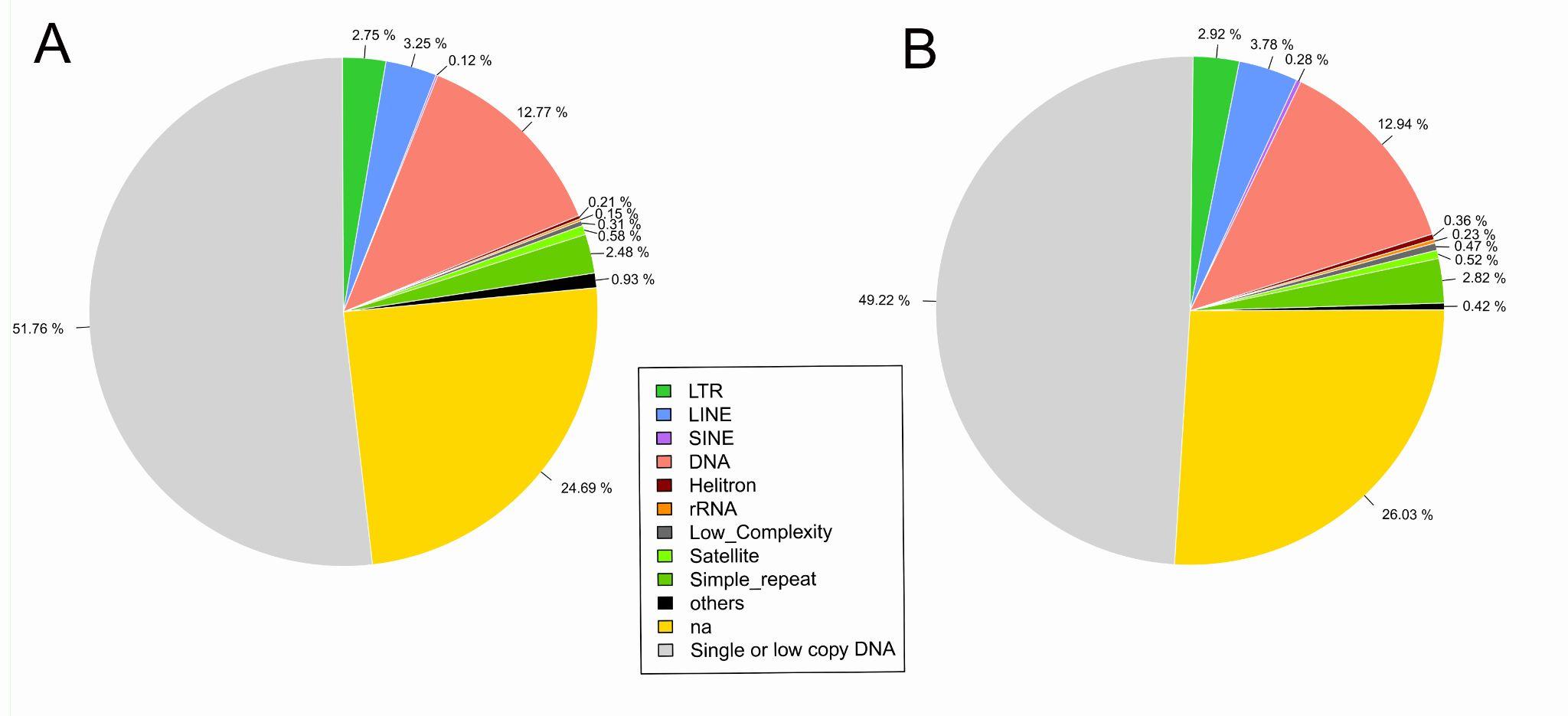


**Supplementary Figure 5:** Piechart showing the repetitive content in *Leptodactylus pentadactylus* male (A) and female (B) sequences using DNApipeTE, as described in **Supplementary figure 11.** “na” indicates non-annotated sequence types, which constitute about one quarter of the repeats inferred.

**
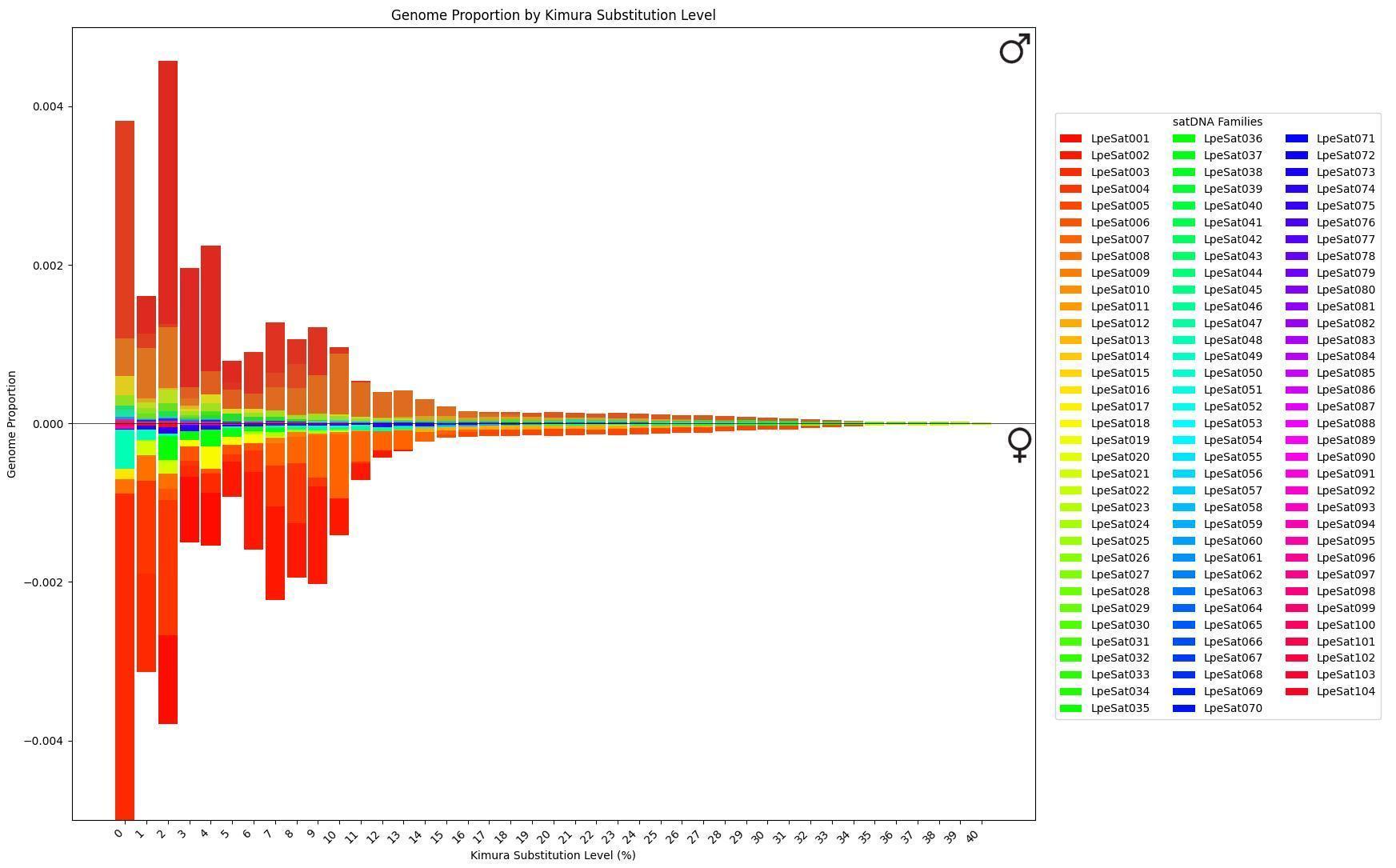
**

**Supplementary Figure 6:** Repeat landscape showing the genome abundance and divergence (Kimura substitution level) of LpeSatDNAs identified in male and female genomes of LPE.

**
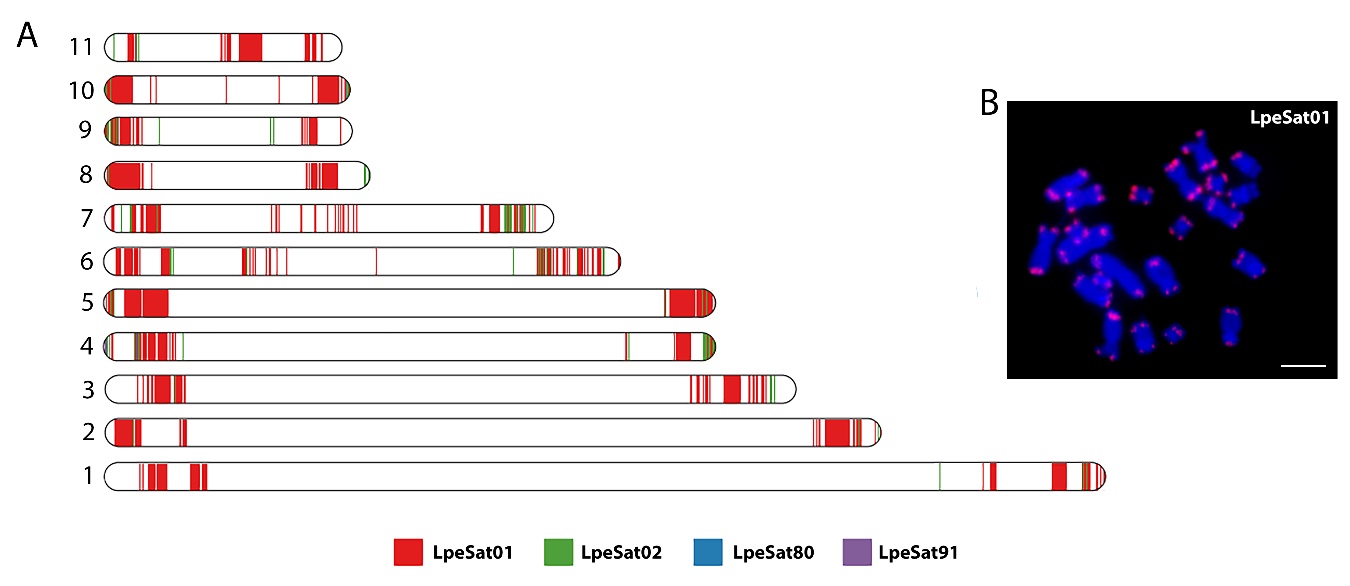
**

**Supplementary Figure 7:** A) In silico mapping of LpeSatDNAs to the genome of *L. fuscus* (GCA_031893025.1) underscoring the widespread and abundance occurrence of LpeSat01 in the terminal regions of all chromosomes. B) In situ mapping of LpeSat01 in a male metaphase plate of *L. fuscus.* Bar = 10 μm.

**
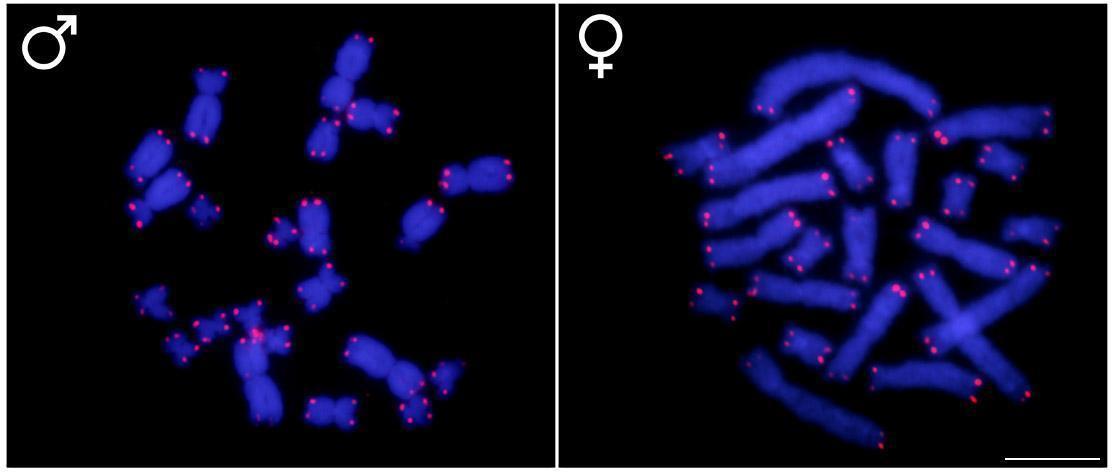
**

**Supplementary Figure 8.** Male and female mitotic cells of *Leptodactylus pentadactylus* hybridized with a telomeric probe. Bar = 10 μm.

**
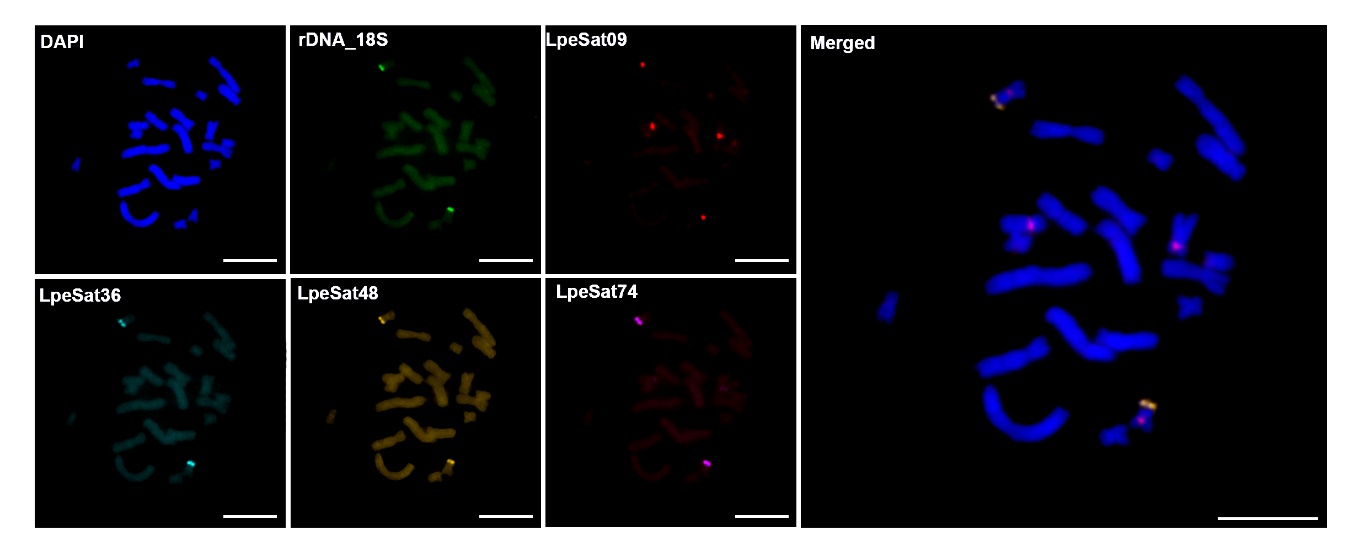
**

**Supplementary Figure 9.** Female mitotic cells from *Leptodactylus pentadactylus* sequentially hybridized with 18S rDNA 18S and several LpeSatDNAs, evidencing their co-localization on the same chromosome (pair 8). Bars = 10 μm.


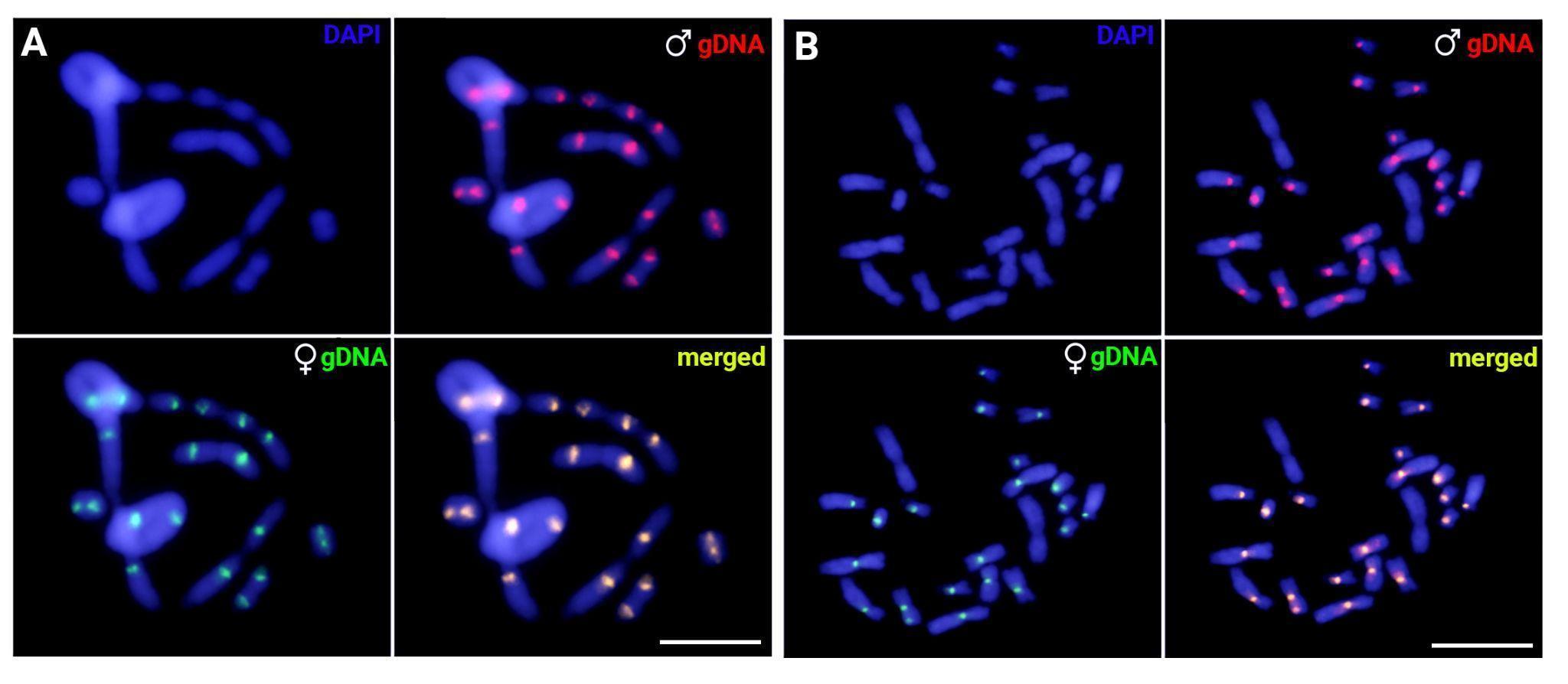


**Supplementary Figure 10.** Male meiotic (A) and mitotic (B) chromosomal preparations of *L. pentadactylus* after intraspecific comparative genomic hybridization (CGH). Chromosome spreads were probed with male- (red) and female-derived (green) genomic probes. The genomic regions with shared hybridization of both probes are yellowish. Scale bar = 10 μm.

**
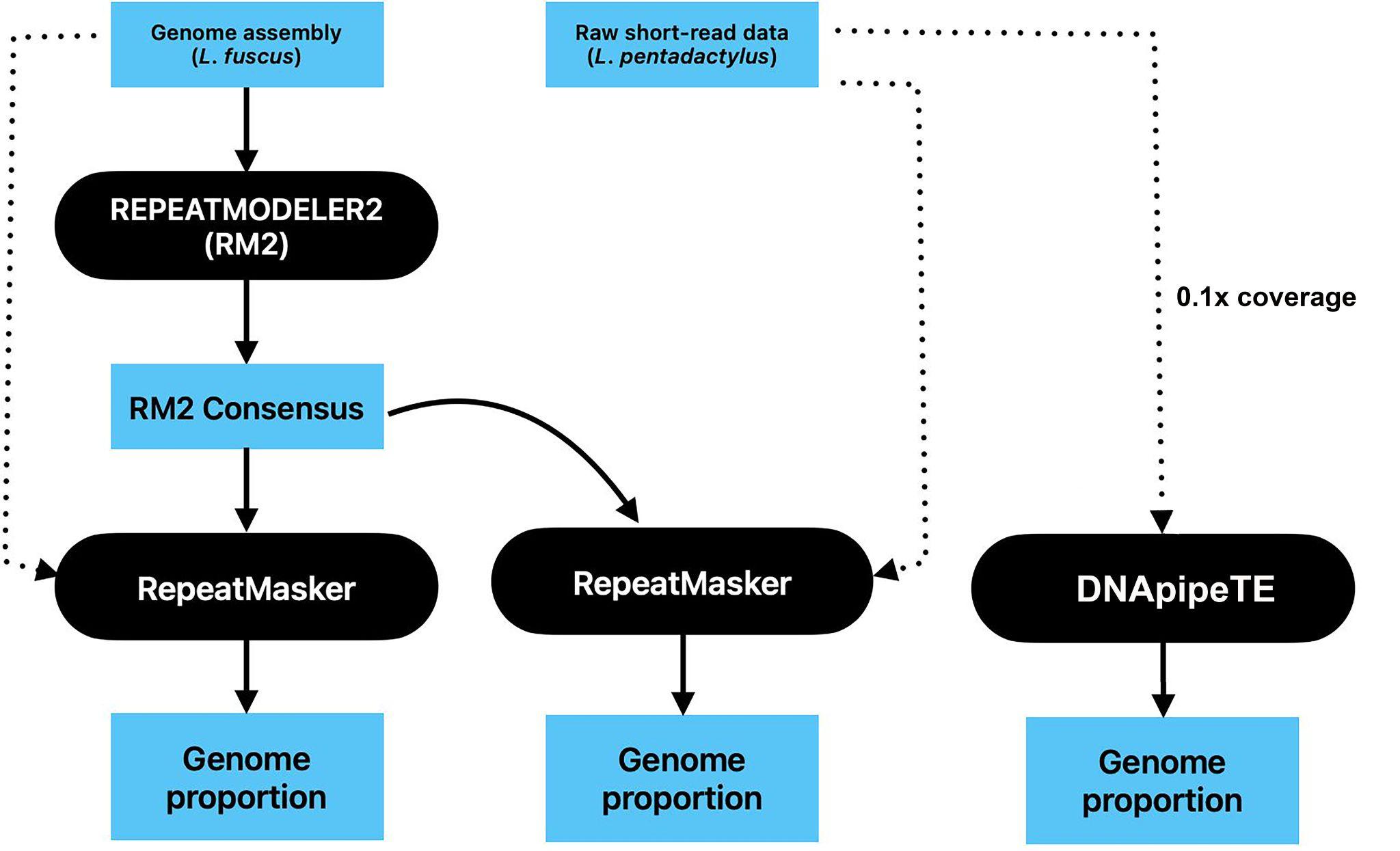
**

**Supplementary Figure 11.** Diagram of the pipeline used to estimate the repetitive contents of *L. fuscus* and *L. pentadactylus*.

**Supplementary Table 1.** Chromosome sizes and morphologies in *L. pentadactylus* (LPE) and *L. fuscus* (LFU). *p* and *q* mean the short and long arms, respectively. See also **Supplementary Figure 2.**

| **Chromosome numbers (ordered by length)** | **LPE** | | | **LFU** | | |
| --- | --- | --- | --- | --- | --- | --- |
|  | **p+q median** | **p median (µm)** | **q median (µm)** | **p+q median (µm)** | **p median (µm)** | **q median (µm)** |
| **1** | 8.10 | 3.49 | 4.61 | 7.80 | 3.50 | 4.30 |
| **2** | 6.90 | 2.52 | 4.38 | 6.00 | 2.40 | 3.60 |
| **3** | 5.90 | 1.80 | 4.10 | 5.20 | 2.10 | 3.10 |
| **4** | 4.77 | 1.05 | 3.72 | 4.82 | 1.06 | 3.76 |
| **5** | 4.60 | 2.00 | 2.60 | 4.68 | 2.30 | 2.38 |
| **6** | 4.20 | 1.60 | 2.60 | 4.48 | 1.28 | 3.20 |
| **7** | 3.20 | 0.90 | 2.30 | 3.46 | 0.96 | 2.50 |
| **8** | 2.86 | 1.26 | 1.60 | 2.67 | 1.19 | 1.48 |
| **9** | 2.34 | 1.00 | 1.34 | 2.06 | 0.86 | 1.20 |
| **10** | 1.98 | 0.94 | 1.04 | 1.97 | 0.89 | 1.08 |
| **11** | 1.88 | 0.84 | 1.04 | 1.77 | 0.80 | 0.97 |

**Supplementary Table 2.** Cytogenetic data for Leptodactylus genera available. The species with meiotic analysis are in bold font. 2n = diploid number; NF = Nombre Fondamental; SCS = Sex chromosome system; MA = Meiotic analyses.

| **Species** | **2n** | **NF** | **SCS** | **MA** | **References** |
| --- | --- | --- | --- | --- | --- |
| *Leptodactylus albilabris* | 22 | 44 | Ukn | NA | ^1^ |
| *Leptodactylus aff bokermanni* | 24 | Ukn | Ukn | **YES** | ^2^ |
| *Leptodactylus bolivianus* | 22 | 44 | Ukn | NA | ^1^ |
| *Leptodactylus bolivianus* | 22 | 44 | Ukn | NA | ^1^ |
| *Leptodactylus bufonius* | 22 | 44 | XY | NA | ^3^ |
| *Leptodactylus cf marmoratus* | **24** | 34 | Ukn | NA | ^2^ |
| *Leptodactylus chaquensis* | 22 | 44 | Ukn | NA | ^1^ |
| *Leptodactylus discodactylus* | 22 | NA | Ukn | NA | ^1^ |
| *Leptodactylus elenae* | 22 | 44 | Ukn | NA | ^1^ |
| *Leptodactylus furnarius* | 22 | 44 | Ukn | NA | ^1^ |
| ***Leptodactylus fuscus*** | **22** | **44** | **Ukn** | **YES** | **^4,^ present study** |
| *Leptodactylus gracilis delattini* | 22 | 44 | Ukn | NA | ^5^ |
| *Leptodactylus gracilis gracilis* | 22 | 44 | Ukn | NA | ^6^ |
| *Leptodactylus gracilis* | 22 | 44 | Ukn | NA | ^1^ |
| *Leptodactylus griseigularis* | 22 | 36 | Ukn | NA | ^1^ |
| *Leptodactylus hylaedactylus* | 26 | Ukn | Ukn | Ukn | ^7^ |
| ***Leptodactylus knudseni*** | 22 | 44 | Ukn | **YES** | ^8^ |
| ***Leptodactylus labyrinthicus*** | **22** | **44** | **Ukn** | **YES** | **^4,^ present study** |
| *Leptodactylus laticeps* | 22 | 44 | Ukn | NA | ^1^ |
| *Leptodactylus latinasus* | 22 | 42 | Ukn | NA | ^1^ |
| ***Leptodactylus latrans*** | **22** | **44** | **Ukn** | **YES** | ^1^**^,^ present study** |
| *Leptodactylus leptodactyloides* | 22 | 36 | Ukn | NA | ^1^ |
| *Leptodactylus macrosternum* | 22 | 42 | Ukn | NA | ^1^ |
| ***Leptodactylus macrosternum*** | 22 | 44 | Ukn | **YES** | ^9^ |
| ***Leptodactylus marmoratus*** | **24** | 34 | Ukn | **YES** | ^2^ |
| *Leptodactylus melanonotus* | 22 | 44 | Ukn | NA | ^2^ |
| *Leptodactylus mystaceus* | 22 | 44 | Ukn | NA | ^1^ |
| ***Leptodactylus mystacinus*** | **22** | **44** | **Ukn** | **YES** | **^4,^ present study** |
| *Leptodactylus natalensis* | 22 | 38 | Ukn | NA | ^1^ |
| ***Leptodactylus ocellatus*** | 22 | Ukn | Ukn | **YES** | ^10^ |
| *Leptodactylus notoaktites* | 22 | 44 | Ukn | NA | ^1^ |
| *Leptodactylus paraensis* | 22 | 44 | **4X4Y** | **YES** | **present study** |
| ***Leptodactylus pentadactylus*** | 22 | Ukn | **5X5Y** | **YES** | ^11,^ **present study** |
| ***Leptodactylus pentadactylus*** | 22 | 44 | **6X6Y** | **YES** | ^12^ |
| *Leptodactylus petersii* | 22 | 42 | Ukn | NA | ^1^ |
| *Leptodactylus petersii* | 22 | 44 | Ukn | NA | ^13^ |
| *Leptodactylus plaumanni* | 22 | 44 | Ukn | NA | ^6^ |
| *Leptodactylus podicipinus* | 22 | 34 | Ukn | NA | ^1^ |
| ***Leptodactylus aff. podicipinus*** | **20** | Ukn | Ukn | **YES** | ^13^ |
| ***Leptodactylus podicipinus*** | 22 | 36 | Ukn | **YES** | ^8^ |
| *Leptodactylus pustulatus* | 22 | 44 | Ukn | NA | ^1^ |
| *Leptodactylus rhodomystax* | 22 | 44 | Ukn | NA | ^13^ |
| *Leptodactylus rhodonotus* | 22 | 44 | Ukn | NA | ^1^ |
| *Leptodactylus riveroi* | 22 | 42 | Ukn | NA | ^1^ |
| *Leptodactylus silvanimbus* | **24** | 48 | Ukn | NA | ^1^ |
| *Leptodactylus syphax* | 22 | 44 | Ukn | NA | ^1^ |
| *Leptodactylus troglodytes* | 22 | 44 | Ukn | NA | ^1^ |
| *Leptodactylus wagneri* | 22 | 36 | Ukn | NA | ^10^ |

**Supplementary Table 3**. General features of *Leptodactylus pentadactylus* satellitome*.* The LpeSatDNA selected for FISH experiments are highlighted in bold. *RUL = Repeat Unit Length”

|  |  |  | **Abundance** | | |  |
| --- | --- | --- | --- | --- | --- | --- |
| **LpeSatDNA family** | **RUL** | **A+T%** | **Male** | **Female** | **M/F** | **Divergence** |
| **LpeSat01** | 35 | 71% | 0.014179 | 0.010638 | 1.332854353 | 3.87 |
| **LpeSat02** | 35 | 69% | 0.008699 | 0.013602 | 0.6395610634 | 9.55 |
| **LpeSat03** | 152 | 55% | 0.007955 | 0.02496 | 0.3187126625 | 2.08 |
| **LpeSat04** | 92 | 42% | 0.007118 | 0.012426 | 0.5727808764 | 6.79 |
| **LpeSat05** | 2216 | 51% | 0.005826 | 0.006415 | 0.9081191438 | 13.51 |
| **LpeSat06** | 2971 | 57% | 0.005164 | 0.005008 | 1.031035915 | 5.66 |
| LpeSat07 | 21 | 57% | 0.005132 | 0.004889 | 1.049613982 | 12.01 |
| **LpeSat08** | 97 | 58% | 0.004455 | 0.003079 | 1.44691215 | 2.98 |
| **LpeSat09** | 1589 | 58% | 0.003188 | 0.00391 | 0.8153829727 | 9.27 |
| **LpeSat10** | 5228 | 56% | 0.002457 | 0.002556 | 0.9611619277 | 11.02 |
| LpeSat11 | 4382 | 46% | 0.00228 | 0.001498 | 1.522164097 | 8.45 |
| LpeSat12 | 1205 | 60% | 0.002058 | 0.002235 | 0.9205658728 | 16.02 |
| LpeSat13 | 36 | 56% | 0.002024 | 0.001383 | 1.463472128 | 3.31 |
| LpeSat14 | 49 | 49% | 0.00191 | 0.001656 | 1.153429878 | 5.50 |
| LpeSat15 | 31 | 58% | 0.001763 | 0.001317 | 1.339149225 | 11.82 |
| LpeSat16 | 6930 | 56% | 0.00165 | 0.001636 | 1.00835934 | 5.35 |
| LpeSat17 | 577 | 57% | 0.00154 | 0.001733 | 0.8882055061 | 6.74 |
| LpeSat18 | 32 | 72% | 0.00145 | 0.002118 | 0.6844769905 | 5.63 |
| LpeSat19 | 44 | 59% | 0.001436 | 0.000774 | 1.855346071 | 4.26 |
| LpeSat20 | 31 | 71% | 0.001436 | 0.001457 | 0.9852910234 | 7.85 |
| LpeSat21 | 568 | 64% | 0.001335 | 0.001878 | 0.7107553132 | 2.70 |
| LpeSat22 | 4743 | 55% | 0.001318 | 0.001541 | 0.854832436 | 13.28 |
| LpeSat23 | 48 | 65% | 0.001251 | 0.001003 | 1.247060041 | 5.80 |
| LpeSat24 | 46 | 61% | 0.001248 | 0.00129 | 0.9676006196 | 8.43 |
| LpeSat25 | 72 | 60% | 0.000995 | 0.001011 | 0.9837593919 | 3.16 |
| LpeSat26 | 42 | 62% | 0.000905 | 0.00069 | 1.31153141 | 6.98 |
| LpeSat27 | 37 | 54% | 0.000814 | 0.000573 | 1.422371809 | 3.49 |
| LpeSat28 | 66 | 52% | 0.000806 | 0.001002 | 0.8048583727 | 4.17 |
| LpeSat29 | 37 | 62% | 0.000772 | 0.000765 | 1.009605763 | 3.23 |
| LpeSat30 | 39 | 56% | 0.000754 | 0.000734 | 1.026927731 | 6.75 |
| LpeSat31 | 84 | 64% | 0.000753 | 0.000558 | 1.349843164 | 18.62 |
| LpeSat32 | 39 | 56% | 0.00064 | 0.000362 | 1.768237995 | 7.17 |
| LpeSat33 | 1038 | 62% | 0.000625 | 0.000774 | 0.8067532999 | 22.09 |
| LpeSat34 | 31 | 61% | 0.00061 | 0.000282 | 2.16119492 | 9.10 |
| LpeSat35 | 50 | 70% | 0.000608 | 0.000495 | 1.226412222 | 7.89 |
| **LpeSat36** | 325 | 29% | 0.000587 | 0.001868 | 0.3142772489 | 4.87 |
| LpeSat37 | 72 | 49% | 0.000557 | 0.000722 | 0.7711051633 | 7.31 |
| LpeSat38 | 41 | 59% | 0.000551 | 0.000383 | 1.439617668 | 12.01 |
| LpeSat39 | 76 | 61% | 0.000547 | 0.000483 | 1.133230926 | 6.74 |
| LpeSat40 | 72 | 56% | 0.00054 | 0.000442 | 1.223167879 | 4.74 |
| LpeSat41 | 1677 | 55% | 0.000517 | 0.000523 | 0.9883594101 | 15.61 |
| LpeSat42 | 605 | 60% | 0.00045 | 0.000447 | 1.008096501 | 22.63 |
| LpeSat43 | 123 | 47% | 0.000433 | 0.000569 | 0.7617489005 | 3.16 |
| LpeSat44 | 38 | 61% | 0.000395 | 0.000297 | 1.329992235 | 9.51 |
| LpeSat45 | 38 | 47% | 0.000392 | 0.000222 | 1.766613324 | 19.93 |
| **LpeSat46** | 108 | 33% | 0.000389 | 0.001256 | 0.3100862798 | 7.68 |
| LpeSat47 | 45 | 60% | 0.000379 | 0.000366 | 1.03409692 | 3.07 |
| **LpeSat48** | 178 | 33% | 0.000377 | 0.001352 | 0.2786608842 | 3.41 |
| LpeSat49 | 30 | 50% | 0.000377 | 0.000338 | 1.113195324 | 15.97 |
| LpeSat50 | 100 | 59% | 0.000338 | 0.000295 | 1.145452364 | 8.38 |
| LpeSat51 | 53 | 83% | 0.000335 | 0.000363 | 0.9235502703 | 12.79 |
| LpeSat52 | 30 | 57% | 0.00032 | 0.000257 | 1.243431614 | 12.91 |
| LpeSat53 | 383 | 63% | 0.00032 | 0.000238 | 1.343617226 | 7.07 |
| LpeSat54 | 99 | 54% | 0.000313 | 0.000278 | 1.124764553 | 6.81 |
| LpeSat55 | 31 | 52% | 0.000307 | 0.00025 | 1.229398627 | 10.47 |
| LpeSat56 | 45 | 67% | 0.000299 | 0.000356 | 0.8393590292 | 8.77 |
| LpeSat57 | 447 | 50% | 0.000293 | 0.000295 | 0.9934053866 | 12.01 |
| LpeSat58 | 30 | 43% | 0.000292 | 0.000177 | 1.648377147 | 5.57 |
| LpeSat59 | 37 | 59% | 0.000275 | 0.000418 | 0.6591347473 | 13.86 |
| LpeSat60 | 98 | 42% | 0.000266 | 0.000144 | 1.843022583 | 2.44 |
| LpeSat61 | 50 | 50% | 0.000255 | 0.000276 | 0.9245201567 | 4.44 |
| LpeSat62 | 42 | 50% | 0.000254 | 0.000255 | 0.9968065582 | 4.16 |
| LpeSat63 | 50 | 64% | 0.000251 | 0.000089 | 2.832278541 | 7.12 |
| LpeSat64 | 35 | 54% | 0.000242 | 0.000203 | 1.187861467 | 6.34 |
| LpeSat65 | 90 | 47% | 0.000232 | 0.000188 | 1.23473886 | 8.23 |
| LpeSat66 | 35 | 71% | 0.000227 | 0.000131 | 1.728533864 | 7.86 |
| LpeSat67 | 465 | 59% | 0.000217 | 0.000218 | 0.996588265 | 14.02 |
| LpeSat68 | 59 | 54% | 0.000216 | 0.000229 | 0.9400255048 | 8.77 |
| LpeSat69 | 38 | 50% | 0.000209 | 0.000198 | 1.05527617 | 8.16 |
| LpeSat70 | 30 | 50% | 0.000209 | 0.000286 | 0.7303831472 | 7.88 |
| LpeSat71 | 141 | 56% | 0.000204 | 0.000148 | 1.381412928 | 3.07 |
| **LpeSat72** | 70 | 53% | 0.000197 | 0.000097 | 2.035481152 | 9.45 |
| LpeSat73 | 46 | 59% | 0.000189 | 0.000142 | 1.333895907 | 5.73 |
| **LpeSat74** | 403 | 31% | 0.000179 | 0.000886 | 0.2016184388 | 7.04 |
| LpeSat75 | 67 | 61% | 0.000157 | 0.000075 | 2.089403001 | 2.42 |
| LpeSat76 | 112 | 65% | 0.000155 | 0.000113 | 1.379621441 | 4.42 |
| LpeSat77 | 41 | 46% | 0.000153 | 0.000183 | 0.8348121312 | 10.56 |
| LpeSat78 | 743 | 54% | 0.000146 | 0.000138 | 1.064089941 | 10.16 |
| LpeSat79 | 30 | 63% | 0.000143 | 0.000104 | 1.368710027 | 9.02 |
| **LpeSat80** | 22 | 50% | 0.000137 | 0.000055 | 2.480094189 | 9.68 |
| LpeSat81 | 35 | 66% | 0.00013 | 0.000124 | 1.048489964 | 6.05 |
| LpeSat82 | 830 | 56% | 0.000129 | 0.00009 | 1.436744307 | 4.24 |
| LpeSat83 | 40 | 68% | 0.000128 | 0.000075 | 1.695355365 | 9.16 |
| LpeSat84 | 27 | 59% | 0.000125 | 0.000077 | 1.63492693 | 7.61 |
| LpeSat85 | 38 | 55% | 0.000124 | 0.000097 | 1.287569341 | 9.23 |
| LpeSat86 | 46 | 54% | 0.000124 | 0.000124 | 0.9957757103 | 4.77 |
| LpeSat87 | 57 | 21% | 0.000117 | 0.000113 | 1.034625585 | 3.03 |
| LpeSat88 | 40 | 48% | 0.000116 | 0.000129 | 0.9012309023 | 5.36 |
| LpeSat89 | 31 | 77% | 0.000114 | 0.000139 | 0.8226507977 | 6.90 |
| LpeSat90 | 101 | 49% | 0.000112 | 0.000077 | 1.462881811 | 4.47 |
| **LpeSat91** | 37 | 59% | 0.000106 | 0.000045 | 2.35004712 | 11.83 |
| LpeSat92 | 217 | 65% | 0.000106 | 0.000123 | 0.8586067519 | 5.29 |
| LpeSat93 | 585 | 62% | 0.000099 | 0.000155 | 0.6417960256 | 4.90 |
| LpeSat94 | 841 | 52% | 0.000098 | 0.000109 | 0.9013428882 | 2.01 |
| LpeSat95 | 45 | 62% | 0.000097 | 0.000071 | 1.367980281 | 9.87 |
| **LpeSat96** | 32 | 38% | 0.000095 | 0.000033 | 2.893865195 | 3.76 |
| LpeSat97 | 615 | 60% | 0.000084 | 0.000095 | 0.8856308975 | 4.05 |
| LpeSat98 | 26 | 62% | 0.000083 | 0.000092 | 0.8963277193 | 4.07 |
| LpeSat99 | 34 | 53% | 0.000083 | 0.000105 | 0.7885979334 | 8.73 |
| **LpeSat100** | 390 | 58% | 0.000078 | 0.000014 | 5.386677474 | 4.57 |
| **LpeSat101** | 74 | 54% | 0.000077 | 0.000011 | 7.327873404 | 4.40 |
| LpeSat102 | 216 | 45% | 0.00007 | 0.00008 | 0.8805428921 | 6.22 |
| LpeSat103 | 56 | 57% | 0.000069 | 0.000122 | 0.5658512868 | 3.29 |
| LpeSat104 | 208 | 53% | 0.000048 | 0.000042 | 1.151284662 | 11.27 |

**Supplementary Table 4.** Pairwise divergence estimated between the species used to make the phylogenetic tree in **Figure 2.**

|  | ***L. pentadactylus*** | ***E. petersi*** | ***L. myersi*** | ***L. vastus*** | ***L. mystacinus*** | ***L. latrans*** | ***L. labyrinthicus*** | ***L. fuscus*** |
| --- | --- | --- | --- | --- | --- | --- | --- | --- |
| ***E. petersi*** | 0.195 |  |  |  |  |  |  |  |
| ***L. myersi*** | 0.074 | 0.184 |  |  |  |  |  |  |
| ***L. vastus*** | 0.106 | 0.182 | 0.076 |  |  |  |  |  |
| ***L. mystacinus*** | 0.128 | 0.199 | 0.113 | 0.126 |  |  |  |  |
| ***L. latrans*** | 0.176 | 0.209 | 0.146 | 0.146 | 0.135 |  |  |  |
| ***L. labyrinthicus*** | 0.084 | 0.194 | 0.060 | 0.101 | 0.128 | 0.158 |  |  |
| ***L. fuscus*** | 0.138 | 0.203 | 0.141 | 0.144 | 0.083 | 0.138 | 0.139 |  |
| ***L. paraensis*** | 0.109 | 0.186 | 0.077 | 0.002 | 0.129 | 0.148 | 0.102 | 0.148 |

**Supplementary Table 5**. List of primers used for the amplification of selected LpeSatDNAs.

| **LpeSatDNAs** | **Forward primer** | **Reverse primer** |
| --- | --- | --- |
| LpeSat03-152 | TGAACTACGCGAAATGTGAT | AACTTCTCCGACTAGGGT |
| LpeSat04-92 | GTCACTGGGCACTAAACG | ATTAACGTGGCAATAACGCA |
| LpeSat05-2216 | ACTCAGGCAACCAGGTAT | TGCACAACCACAGACACT |
| LpeSat06-2971 | CATATCGGCTGTTGTAGG | ACACTATGTATGAACTGTGA |
| LpeSat08-97 | TCAGGATCAGTACAGGATA | TTATACTCCAGAGCTGCG |
| LpeSat09-1589 | TATGTGTCTGCTTCTGAGC | GACGGTTGGCAGAAGAAAT |
| LpeSat10-5228 | AAGCACCTGTCAGATGAGA | TGAGGATAGCATGGATGTC |
| LpeSat36-325 | AGCCAGAGATGACCGTGA | TCTGGCTACGAGGCTACT |
| LpeSat46-108 | TGCAGCTTGGCTTCACCC | CATCAATCCCAGGAAAGGG |
| LpeSat48-178 | TTAGAGCTGCATTTGCCC | AGAGCAAGAGCGCAGAGA |
| LpeSat72-70 | TCCCTTTGCCCACTAGAA | TGGTCCTTTAGCCCACTA |
| LpeSat74-403 | AGAATGCCAGACCTCGCT | TGACATCCTGTGTCTTCTTT |
| LpeSat100-390 | ACGTCGACTCATTGGAAG | GGGAAGTAAGGTGTTGGT |
| LpeSat101-74 | TACTACACAGACAGGACCA | CGTGCGGACATATTGTGT |

**Supplementary References**

1. Coelho, A. C. et al. Intra-generic and interspecific karyotype patterns of *Leptodactylus* and *Adenomera* (Anura, Leptodactylidae) with inclusion of five species from Central Amazonia. *Genetica* **144**, 37–46 (2016).

2. Campos, J. R. C. et al. Chromosome evolution in three Brazilian *Leptodactylus* species (Anura, Leptodactylidae), with phylogenetic considerations. *Hereditas* **146**, 104–111 (2009)

3. Schneider, R. G. et al. Sex chromosomes in the Vizcacheras’ White-lipped frog, *Leptodactylus bufonius* (Anura, Leptodactylidae). *Acad. Bras. Ciênc.* **93**, e20190426 (2021)

4. Silva, A. P., Haddad, C. F. & Kasahara, S. Chromosomal studies on five species of the genus *Leptodactylus* Fitzinger, 1826 (Amphibia, Anura) using differential staining. *Cytobios* **103**, 25–38 (2000)

5. Gazoni, T. et al. Chromosome mapping of U2 snDNA in species of *Leptodactylus* (Anura, Leptodactylidae). *Cytogenet Genome Res.* **161**, 63–69 (2021)

6. Silva, A. P. Z., Garcia, P. C. A., Martins, V. G., Bacci, M. & Kasahara, S. Chromosomal and molecular analyses of *Leptodactylus gracilis gracilis*, *L. gracilis delattini*, and *L. plaumanni* (Anura, Leptodactylidae): taxonomic implications. *Amphibia-Reptilia* **25**, 185–196 (2004)

7. Heyer, W. R., & Diment, J. M. The karyotype of *Vanzolinius discodactylus* and comments on usefulness of karyotypes in determining relationships in the Leptodactylus-complex (Amphibia, Leptodactylidae). *Proc. Biol. Soc. Wash*. 87(29), 327-336 (1974)

8. Amaro-Ghilardi, R. C., Rodrigues, M. T. & Yonenaga-Yassuda, Y. Chromosomal studies after differential staining and fluorescence in situ hybridization using telomeric probe in three *Leptodactylus* species (Leptodactylidae, Anura). *Caryologia* (2004)

9. da Silva, D. S., da Silva Filho, H. F., Cioffi, M. B., de Oliveira, E. H. C. & Gomes, A. J. B. Comparative cytogenetics in four *Leptodactylus* species (Amphibia, Anura, Leptodactylidae): evidence of inner chromosomal diversification in highly conserved karyotypes. *Cytogenet. Genome Res.* **161**, 52–62 (2021)

10. King, M. The evolution of heterochromatin in the amphibian genome. In: Green, D. M., Sessions, S. K. (eds) Amphibian cytogenetics and evolution, 359–391 (Academic Press, 1991).

11. Noronha, R. C. R. et al. Meiotic analyses show adaptations to maintenance of fertility in X1Y1X2Y2X3Y3X4Y4X5Y5 system of Amazon frog *Leptodactylus pentadactylus* (Laurenti, 1768). *Sci. Rep.* **10**, 1–13 (2020)

12. Gazoni, T. et al. More sex chromosomes than autosomes in the Amazonian frog *Leptodactylus pentadactylus*. *Chromosoma* **127**, 269–278 (2018)

13. Gazoni, T. et al. Cytogenetic analyses of eight species in the genus *Leptodactylus* Fitzinger, 1843 (Amphibia, Anura, Leptodactylidae), including a new diploid number and a karyotype with multiple translocations. *BMC Genet.* **13**, 1–16 (2012)
