# Supplementary material for: Investigating the evolution of large meiotic rings of multiple X and Y sex chromosomes in two Leptodactylus frog species (Anura, Leptodactylidae)
Source: Commun Biol. 2025 Nov 21;8:1636. doi: 10.1038/s42003-025-09151-z (PMC12638755; doi:10.1038/s42003-025-09151-z)
Supplement: Supplementary file 2 — Description of Additional Supplementary Materials [file 42003_2025_9151_MOESM2_ESM.pdf]

Description of Additional Supplementary Files

File name: Supplementary Data

Description: Numerical source data for the graph presented in Figure 4
