# Supplementary material for: Investigating the evolution of large meiotic rings of multiple X and Y sex chromosomes in two Leptodactylus frog species (Anura, Leptodactylidae)
Source: Commun Biol. 2025 Nov 21;8:1636. doi: 10.1038/s42003-025-09151-z (PMC12638755; doi:10.1038/s42003-025-09151-z)
Supplement: Supplementary file 4 — Reporting Summary [file 42003_2025_9151_MOESM4_ESM.pdf]

nature portfolio

Corresponding author(s): Thomas Liehr

Last updated by author(s): Oct 12, 2025

Reporting Summary

Nature Portfolio wishes to improve the reproducibility of the work that we publish. This form provides structure for consistency and transparency in reporting. For further information on Nature Portfolio policies, see our Editorial Policies and the Editorial Policy Checklist.

Statistics

For all statistical analyses, confirm that the following items are present in the figure legend, table legend, main text, or Methods section.

n/a	Confirmed	
☐	☒	The exact sample size (*n*) for each experimental group/condition, given as a discrete number and unit of measurement
☐	☒	A statement on whether measurements were taken from distinct samples or whether the same sample was measured repeatedly
☐	☒	The statistical test(s) used AND whether they are one- or two-sided<br>*Only common tests should be described solely by name; describe more complex techniques in the Methods section.*
☐	☒	A description of all covariates tested
☐	☒	A description of any assumptions or corrections, such as tests of normality and adjustment for multiple comparisons
☐	☒	A full description of the statistical parameters including central tendency (e.g. means) or other basic estimates (e.g. regression coefficient) AND variation (e.g. standard deviation) or associated estimates of uncertainty (e.g. confidence intervals)
☐	☒	For null hypothesis testing, the test statistic (e.g. *F*, *t*, *r*) with confidence intervals, effect sizes, degrees of freedom and *P* value noted<br>*Give P values as exact values whenever suitable.*
☒	☐	For Bayesian analysis, information on the choice of priors and Markov chain Monte Carlo settings
☒	☐	For hierarchical and complex designs, identification of the appropriate level for tests and full reporting of outcomes
☒	☐	Estimates of effect sizes (e.g. Cohen's *d*, Pearson's *r*), indicating how they were calculated

Our web collection on statistics for biologists contains articles on many of the points above.

Software and code

Policy information about availability of computer code

Data collection	n/a
Data analysis	https://github.com/fjruizruano/satminer; https://github.com/fjruizruano/ngs-protocols/blob/master/mapping_blat_gs.py; QGIS 3.32 (Lima); ISIS (MetaSystems Hard & Software GmbH, Altlussheim, Germany); Natural Earth (https://www.naturalearthdata.com/).

For manuscripts utilizing custom algorithms or software that are central to the research but not yet described in published literature, software must be made available to editors and reviewers. We strongly encourage code deposition in a community repository (e.g. GitHub). See the Nature Portfolio guidelines for submitting code & software for further information.

Data

Policy information about availability of data

All manuscripts must include a data availability statement. This statement should provide the following information, where applicable:

- Accession codes, unique identifiers, or web links for publicly available datasets
- A description of any restrictions on data availability
- For clinical datasets or third party data, please ensure that the statement adheres to our policy

Sequencing data that support the findings of this study have been deposited
in Sequence Read Archive (SRA-NCBI) under accession numbers SRR30896012 (male) and SRR30896011 (female). Satellites DNA (satDNA) sequences characterized from Leptodactylus pentadactylus individuals are deposited in the GenBank database under accession numbers (PQ462747 - PQ462850). 16S rDNA sequences from

L. pentadactylus, L. mystacinus, L. latrans, L. labyrinthicus, L. fuscus and L. paraensis under access number PX048541, PX048542, PX048543, PX048544, PX048545, PX048546, respectively. (https://www.ncbi.nlm.nih.gov/). All additional data supporting the findings of this study are available within the paper and its Supplementary Information.

Research involving human participants, their data, or biological material

Policy information about studies with human participants or human data. See also policy information about sex, gender (identity/presentation), and sexual orientation and race, ethnicity and racism.

Reporting on sex and gender	n/a
Reporting on race, ethnicity, or other socially relevant groupings	n/a
Population characteristics	n/a
Recruitment	n/a
Ethics oversight	n/a

Note that full information on the approval of the study protocol must also be provided in the manuscript.

Field-specific reporting

Please select the one below that is the best fit for your research. If you are not sure, read the appropriate sections before making your selection.

☒ Life sciences ☐ Behavioural & social sciences ☐ Ecological, evolutionary & environmental sciences

For a reference copy of the document with all sections, see nature.com/documents/nr-reporting-summary-flat.pdf

Life sciences study design

All studies must disclose on these points even when the disclosure is negative.

Sample size	We collected specimens from 6 Leptodactylus species: 1- Leptodactylus pentadactylus (LPE); 2- Leptodactylus paraensis (LPA); 3- Leptodactylus fuscus; 4- Leptodactylus mystacinus; 5- Leptodactylus labyrinthicus; 6- Leptodactylus latrans<br>Chromosomes were successfully obtained for all sampled individuals. Additionally, selected individuals were sent for sequencing. This sample size aligns with the standards of satellitome studies (e.g., Crepaldi & Parise-Maltempi, 2020; Serrano-Freitas et al., 2020; de Oliveira et al., 2023).
Data exclusions	No data were excluded.
Replication	Independent runs of each computational analysis were conducted to ensure the reproducibility and reliability of the results. For each analysis, the procedures were repeated multiple times, using the same input data and parameters, to confirm that the outputs were consistent. In every case, the independent runs returned identical results. All parameters used are included in Methods section of the manuscript.
Randomization	No randomization was performed.
Blinding	The sample IDs of individuals sent for DNA sequencing were not disclosed to the analyst. Chromosome preparation was conducted prior to the taxonomic identification of the samples, ensuring that the process was unbiased by specific species-level information. Satellitome analyses were performed with access restricted to basic metadata, including the sex of the individuals and their broader biological classification (Order).

Reporting for specific materials, systems and methods

We require information from authors about some types of materials, experimental systems and methods used in many studies. Here, indicate whether each material, system or method listed is relevant to your study. If you are not sure if a list item applies to your research, read the appropriate section before selecting a response.

Materials & experimental systems

n/a	Involved in the study
☒	☐ Antibodies
☒	☐ Eukaryotic cell lines
☒	☐ Palaeontology and archaeology
☐	☒ Animals and other organisms
☒	☐ Clinical data
☒	☐ Dual use research of concern
☒	☐ Plants

Methods

n/a	Involved in the study
☒	☐ ChIP-seq
☒	☐ Flow cytometry
☒	☐ MRI-based neuroimaging

Animals and other research organisms

Policy information about studies involving animals; ARRIVE guidelines recommended for reporting animal research, and Sex and Gender in Research

Laboratory animals: n/a

Wild animals: Specimens were collected using a casting net from their original habitat to ensure minimal stress during transport (Authorization ICMBio/SISBIO 96067-1). Upon arrival, the animals were maintained in small boxes under appropriate conditions. For euthanasia, an overdose of anesthesia was administered using clove oil (Eugenol), prepared by diluting it in 95% ethanol to a final concentration of 60 mg/L in water. This procedure was conducted in accordance with the guidelines approved by the Ethics Committee on Animal Experimentation of the Universidade Federal de São Carlos (Process number CEUA 7994170423).

Reporting on sex: Sex was determined by morphology of the gonads and the presence of sex chromosomes confirmed by cytogenetics.

Field-collected samples: n/a

Ethics oversight: Collection of samples was authorized by the Brazilian Environmental Agency Authorization ICMBio/SISBIO 96067-1. Procedures were approved by the Ethics Committee on Animal Experimentation of the Universidade Federal de São Carlos (Process number CEUA 7994170423).

Note that full information on the approval of the study protocol must also be provided in the manuscript.

Plants

Seed stocks: n/a

Novel plant genotypes: n/a

Authentication: n/a
